# Distribution of Holliday junctions and repair forks during *Escherichia coli* DNA double-strand break repair

**DOI:** 10.1371/journal.pgen.1009717

**Published:** 2021-08-25

**Authors:** Tahirah Yasmin, Benura Azeroglu, Charlotte A. Cockram, David R. F. Leach

**Affiliations:** Institute of Cell Biology, School of Biological Sciences, University of Edinburgh, King’s Buildings, Edinburgh, United Kingdom; Universidad de Sevilla, SPAIN

## Abstract

Accurate repair of DNA double-strand breaks (DSBs) is crucial for cell survival and genome integrity. In *Escherichia coli*, DSBs are repaired by homologous recombination (HR), using an undamaged sister chromosome as template. The DNA intermediates of this pathway are expected to be branched molecules that may include 4-way structures termed Holliday junctions (HJs), and 3-way structures such as D-loops and repair forks. Using a tool creating a site-specific, repairable DSB on only one of a pair of replicating sister chromosomes, we have determined how these branched DNA intermediates are distributed across a DNA region that is undergoing DSB repair. In cells, where branch migration and cleavage of HJs are limited by inactivation of the RuvABC complex, HJs and repair forks are principally accumulated within a distance of 12 kb from sites of recombination initiation, known as Chi, on each side of the engineered DSB. These branched DNA structures can even be detected in the region of DNA between the Chi sites flanking the DSB, a DNA segment not expected to be engaged in recombination initiation, and potentially degraded by RecBCD nuclease action. This is observed even in the absence of the branch migration and helicase activities of RuvAB, RadA, RecG, RecQ and PriA. The detection of full-length DNA fragments containing HJs in this central region implies that DSB repair can restore the two intact chromosomes, into which HJs can relocate prior to their resolution. The distribution of recombination intermediates across the 12kb region beyond Chi is altered in *xonA*, *recJ* and *recQ* mutants suggesting that, in the RecBCD pathway of DSB repair, exonuclease I stimulates the formation of repair forks and that RecJQ promotes strand-invasion at a distance from the recombination initiation sites.

## Author summary

DNA double-strand breaks need to be accurately repaired to ensure cell survival. In the bacterium *Escherichia coli*, these breaks are repaired by the RecBCD pathway of homologous recombination, which involves copying genetic information from another intact and identical DNA template. During this repair process, the broken DNA and the template DNA form 4-way joint DNA molecules called Holliday junctions, and information lost from the broken chromosome is restored by DNA replication at three-way repair forks. Here, we have investigated the distribution of Holliday junctions and repair forks relative to a specific DNA double-strand break site in the *E*. *coli* chromosome, in cells where the movement and resolution of joint molecules is limited. Investigation of the impacts of Exonuclease I and the exonuclease-helicase RecJQ on the distribution of these joint molecules has led us to propose that, in the RecBCD pathway of homologous recombination, RecJQ can extend the region of single-stranded DNA on the broken chromosome and Exonuclease I can facilitate the formation of a repair fork by digesting the single-stranded DNA after it has paired with its template.

## Introduction

The repair of DNA double-strand breaks (DSBs) is crucial for cell viability and its accuracy is important to prevent genome rearrangements. Chromosomal DNA replication is one of the major sources of spontaneous DSBs in living cells. Their frequency scales approximately with genome size, so human cells suffer fifty DSBs per S phase [1] and fast-growing *E*. *coli* cells suffer one break in every five generations [2–4]. Homologous recombination (HR) is ideally suited for the accurate repair of replication-dependent DSBs as, following DNA replication, the sister chromosomes are located close to each other. *E*. *coli* uses the HR pathway [5] to repair such damage and aspects of the pathway are conserved from bacteria to humans [6, 7]. The pathway is initiated by RecBCD protein, which acts on DNA double-strand ends and forms 3′ single-stranded overhangs by extensively processing them, following recognition of a Chi sequence [8] during the phase called pre-synapsis. RecBCD then loads the DNA binding protein RecA onto these overhangs [9] and RecA forms nucleoprotein filaments, which, during the synapsis phase, initiate homology search and invade homologous duplex DNA to form 3-way structures called D-loops [5]. These D-loops mature into 4-way DNA structures termed Holliday junctions (HJs) [10] and the two chromosomes now joined by these branched molecules, need to be separated. Therefore, the final post-synaptic phase involves RuvABC protein, which resolves the four-way DNA junctions. Also, DNA synthesis is re-established from the D-loops, initiated by PriA-mediated replication fork restart [11] and the information that was lost as a result of the DSB and the processing of the DNA ends is restored thus directly linking replication to the recombination process. HJs, D-loops and repair forks are all branched molecules that are expected DNA intermediates of this pathway. Due to the short-lived nature of the intermediates of the HR pathway and the overlapping action of the pathway proteins, both the mode and the extent of action of several of the pathway proteins in live cells remain a mystery. Therefore, the structure and the distribution of the late recombination intermediates of the pathway can shed light on the roles of proteins that are key to this pathway.

In this work, we set out to investigate the distribution of 4-way and 3-way intermediates of HR, over a large chromosomal region undergoing repair. Using an inducible palindrome/SbcCD system [12], we were able to introduce and confine a two-ended DSB to only one of the replicating sister chromosomes at a specific site in the chromosomal *lacZ* gene and thereafter investigate the branched recombination intermediates that were generated during the repair of this DSB. HJs can move along DNA by either spontaneous or enzymatically driven branch migration. Therefore, in order to map the HJs to the chromosomal locations where they are generated, their branch migration needed to be restricted and their resolution blocked. In *E*. *coli*, HJs are branch migrated and resolved by a structure-selective endonuclease complex, the RuvABC protein [13, 14] and it is known that Δ*ruvAB* mutant strains accumulate chromosomal HJs when undergoing DSB repair [15]. The spontaneous branch migration activity of the HJs can be restricted by inter-strand DNA crosslinking so that they will not fall off restriction fragments when analysed. In this study, we therefore analysed the distribution pattern of HJs across crosslinked DNA fragments isolated from Δ*ruvAB* mutant strains. We also analysed the distribution of branched molecules in the same DNA fragments in the absence of crosslinking, conditions where 3-way repair forks would be expected to be more stable than 4-way HJs.

We found that RecBCD protein, having recognised a triple Chi array, can initiate recombination events that lead to the formation of HJs and repair forks, most of which are located up to 12 kb away from the Chi site. This distribution of intermediates corresponds well to the region of DNA coated by RecA protein, as detected by ChIP [16, 17] and confirmed here. Surprisingly however, some HJs were detected between the DSB site and Chi, a region of DNA where no RecA protein was detected by ChIP. This implies an unexpected movement of HJs towards the DSB site in a Δ*ruvAB* mutant. We therefore sought to determine whether some other helicase was migrating HJs back towards the DSB site. RecG, an ATPase and 3′-5′ helicase, can bind and dissociate HJs by catalysing branch migration [18]. RecQ, another 3′-5′ DNA helicase, has been found to unwind joint molecules by branch migration [19]. RadA, an ATPase, has been shown to enhance RecA-mediated recombination *in vitro* by stimulating branch migration [20]. In the absence of RecG and RuvAB proteins, the 3′-5′ DNA helicase activity of PriA unwinds the D-loops, thereby destabilising joint molecules [17]. This destabilisation can be suppressed if PriA helicase is inactivated. We reveal that the movement of HJs toward the DSB site still takes place in the absence of all these helicase activities. In the absence of evidence for helicase promoted migration of HJs, we propose that repair forks, moving towards the DSB site, may pull the HJs in their wake through DNA topology. The observation of fully formed DNA fragments containing HJs in the DSB region between the first Chi sites in the genome implies that repair synthesis can occur prior to HJ resolution in a Δ*ruvAB* mutant.

The distribution of recombination intermediates detected in the absence of RuvAB, across the 12kb region beyond Chi, was affected by inactivation of two accessory recombination nucleases: exonuclease I (ExoI) and RecJ, the latter in conjunction with the helicase RecQ. Exonuclease I (ExoI) degrades single-stranded DNA in a 3’-5’ direction whereas RecJ acts in the 5’-3’ direction and in a Δ*recBCD* background, RecQ helicase assists RecJ in catalysing the degradation of the 5′-overhang by unwinding the break ends [21–25]. To our surprise, these results imply that ExoI can play a role in establishing repair forks while RecJQ may promote strand-exchange farther away from the Chi site. These observations have implications for our understanding of the pathway of HR and lead us to propose a model in which synapsis is initiated at any location on the RecA-coated single-strand of DNA to form a paranemic joint whereupon there is a role for post-synaptic 3’-5’ exonuclease action to stimulate the incorporation of a 3’ extremity into the D-loop to enable the formation of a repair fork.

## Results

### System for analysing Holliday junction and repair fork distribution following a replication-dependent DNA double-strand break at the *E*. *coli lacZ* locus

**Fig 1:**
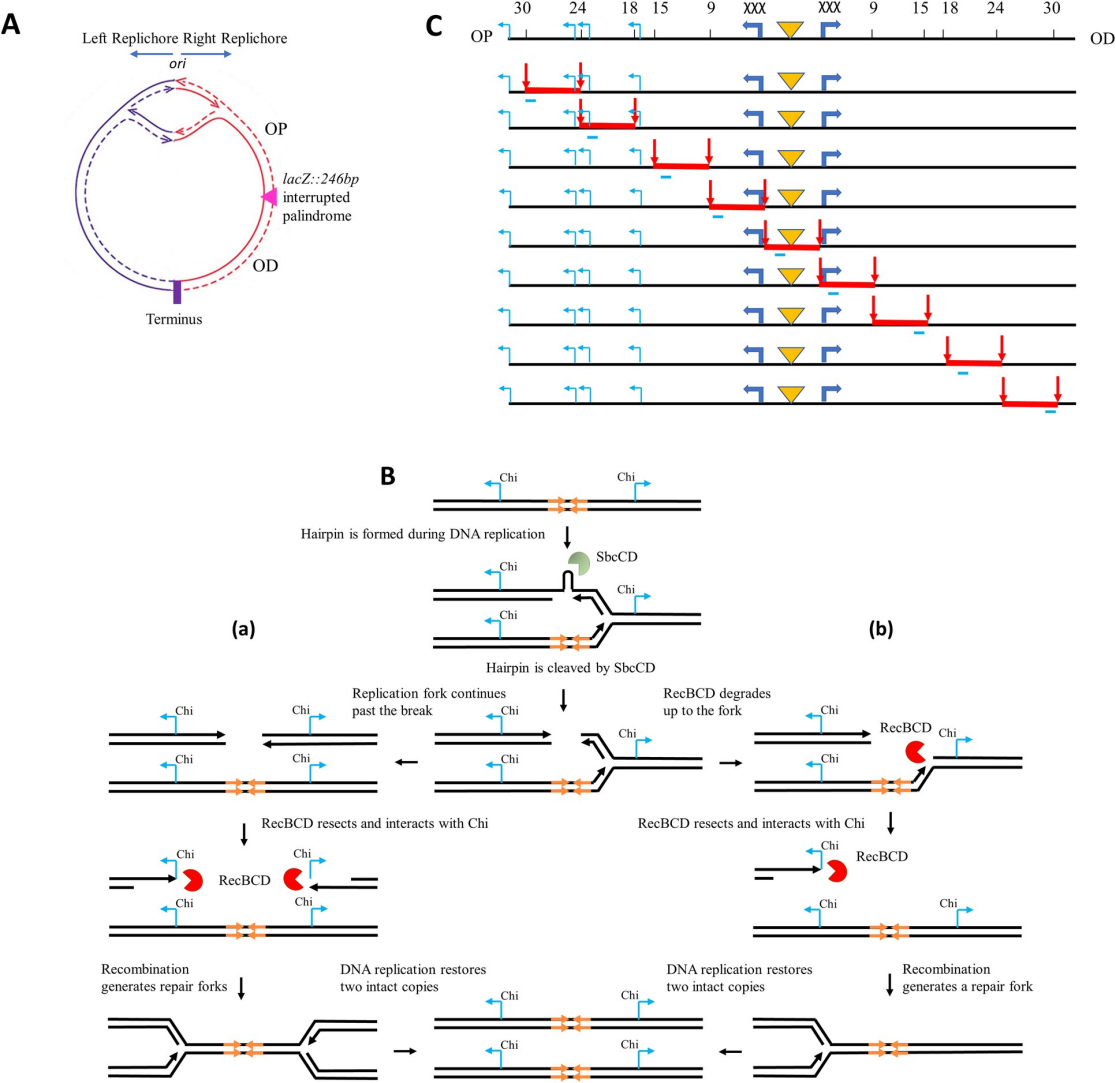
System for analysing Holliday junction and repair fork distribution across a DSB repair region at the *E*. *coli lacZ* locus. A) A schematic representation of *E*. *coli* circular chromosome showing bidirectional replication originating from *oriC* and the location of the palindrome within the *lacZ* region in the right replichore. B) An illustration showing SbcCD-mediated cleavage of the 246bp interrupted palindrome that results in either a two-ended DSB (a) or a one-ended DSB (b). These alternative outcomes are approximately equally probable in a population of cells. The palindrome is highlighted by orange arrows. C) Map of the NotI restriction sites inserted into the DSB region of the *E*. *coli* chromosome. The restriction sites and their distance from the palindrome are marked with black vertical lines and numbers (in kb), respectively on the top line. Below it, the fragments that are generated in 9 different strains following NotI digestion are shown by red lines where the restriction sites are shown by red vertical arrows and the probe binding sites are indicated by light blue bars below the fragments. The palindrome is marked by an orange triangle and the triple Chi arrays and endogenous Chi sites are represented by blue arrows. OP represents origin-proximal side and OD represents origin-distal side.

The distribution of branched intermediates of the HR pathway was studied using a restriction enzyme digestion strategy that resulted in the sampling of a 60kb DSB region with 9 fragments of 6kb in length. These fragments were analysed by two-dimensional (2-D) gel electrophoresis followed by Southern Blotting and probing for the appropriate 6 kb fragment. The DSB was generated at the *lacZ* locus of the *E*. *coli* chromosome by SbcCD-mediated cleavage of a DNA hairpin structure formed by a 246bp interrupted palindrome on the lagging-strand DNA template during replication (Fig 1A and 1B) [12]. The induction of the DSB was controlled by placing the SbcCD endonuclease under an arabinose inducible promoter. The repair intermediates were accumulated and analysed in Δ*ruvAB* mutant strains, which are unable to catalytically branch migrate and resolve Holliday junctions [13,14] and in Δ*ruvAB* mutant strains carrying further mutations of interest. The spontaneous branch migration activity of HJs was restricted by inter-strand DNA crosslinking using trimethylpsoralen (TMP) so that HJs could not fall off the restriction fragments analysed. Since TMP crosslinking mainly targets 5´-TpA dinucleotides of duplex DNA [26], restriction enzyme digestion, with enzymes where the recognition site contains a TA dinucleotide, may be hindered by crosslinking. Therefore, the restriction enzyme NotI was selected to digest the DSB region since the NotI cutting site only consists of GC base pairs.

Twelve restriction sites for NotI were designed to be introduced within the DSB region, six on either side of the palindrome (DSB site) that would generate nine different fragments (Fig 1C). The cutting sites were inserted in pairs in each strain, so that digestion using NotI would generate one 6kb fragment in an individual strain. The fragment length was critically maintained to be similar in order to avoid any effect of varied fragment lengths on the distinct migration pattern of the different branched intermediates generated and accumulated in these fragments. Fig 1C shows the locations of all the artificially inserted NotI restriction sites in this chromosomal region and their relative distances from the DSB site. In this system, a synthetic array of three Chi sites was introduced at 3kb on either side of the DSB. The reason behind inserting three Chi sites instead of one Chi lies in the fact that arrays of three synthetic Chi sites have been shown to be recognized by RecBCD with an efficiency of 60–80% whereas a single Chi site is recognized by RecBCD with an efficiency of only 20–40% [27, 28]. Two of the NotI cutting sites were inserted near the triple Chi arrays on both origin-proximal and origin-distal sides, between the palindrome and the Chi array. Since Chi is the recombination initiation site where RecBCD protein switches its conformation and initiates recombination [29], and no Chi site was present between these two restriction sites, the DSB central fragment was designed to be devoid of any recombination initiation event. On each side of the DSB, four more fragments were designed starting from the Chi arrays and ending 30kb away from the DSB site (3kb-9kb, 9kb-15kb, 18kb-24kb and 24kb-30kb from the DSB site). All the target sites were inserted into intergenic regions or in nonessential genes.

### Distribution of Holliday junctions across the DSB region in Δ*ruvAB* mutants reveals that intermediates mainly form across 30kb around the DSB and up to 12kb from the initiating Chi site

**Fig 2:**
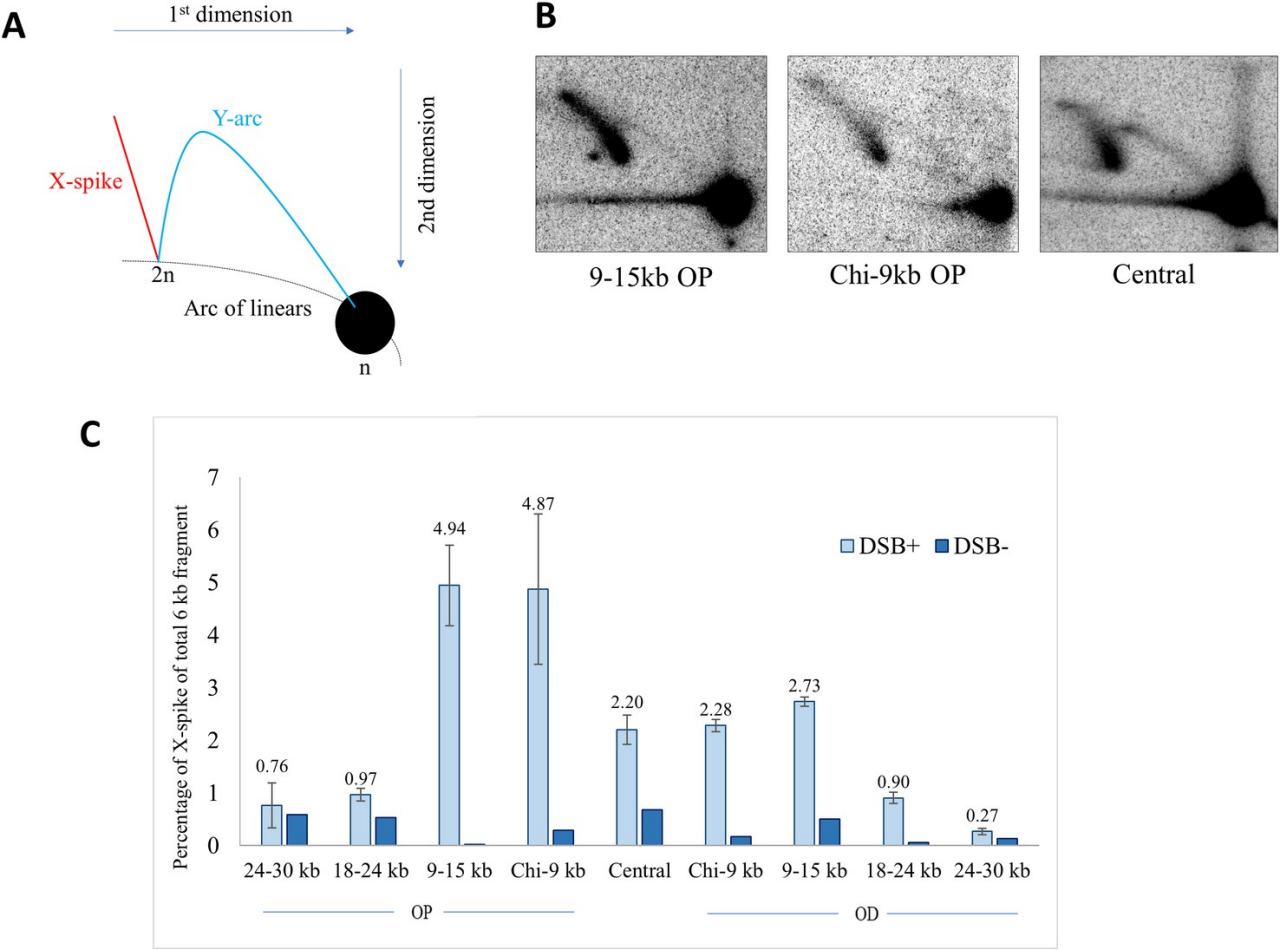
Distribution of Holliday junction DNA structures across the *lacZ* region of Δ*ruvAB* mutant strains as a function of induction of DSB repair. A) Schematic diagram of a native-native 2-D electrophoresis gel showing the expected migration pattern of 3-way (Y-arc) and 4-way (X-spike) DNA structures denoted by blue and red lines, respectively. n represents linear, un-replicated DNA while 2n represents linear, replicated DNA. B) 2-D gels of the crosslinked 9-15kb OP, Chi-9kb OP and central fragments for Δ*ruvAB* strains (DL7259, DL7253 and DL7272, respectively) containing the palindrome, grown in the presence of 0.2% arabinose for 60 minutes. C) Quantification of the signal intensities of HJs generated across the 60kb region following a DSB. The HJ quantification was done on crosslinked DNA samples and is presented as a percentage of X-spike out of the total 6kb DNA fragment (DSB^+^). The quantification of X-spikes from control blots (2-D gel analysis of crosslinked DNA fragments isolated from cell cultures grown in presence of glucose to repress the expression of *sbcDC* is also shown (DSB^-^). The strains used were DL7271 (Δ*ruvAB* 24-30kb OP), DL7270 (Δ*ruvAB* 18-24kb OP), DL7259 (Δ*ruvAB* 9-15kb OP), DL7253(Δ*ruvAB* Chi-9kb OP), DL7272 (Δ*ruvAB* Chi-Chi), DL7251 (Δ*ruvAB* Chi-9kb OD), DL7258 (Δ*ruvAB* 9-15kb OD), DL7261 (Δ*ruvAB* 18-24kb OD) and DL7262 (Δ*ruvAB* 24-30kb OD). OP and OD represent origin-proximal and origin-distal sides. Error bars represent the standard error of the mean where the number of replicates is 3 for the DSB^+^ strains. The experiments on DSB^-^ strains were done once.

The accumulation of HJs within the 6 kb fragments was determined by 2-D gel electrophoresis analysis, a useful technique for distinguishing between 4-way DNA junctions and 3-way DNA junctions that are represented by X-spike and Y-arc migration patterns, respectively (Fig 2A). Representative examples of Southern blots for the central fragment and the immediate next two fragments, located between the Chi array and 15kb away from the palindrome on the origin-proximal side, are shown in Fig 2B. Representative examples of Southern blots for the remaining fragments and all the control blots are shown in S1A and S1B Fig. Fig 2C shows the percentage of HJ hybridisation out of the total hybridisation across the 9 fragments aligned from the fragment most proximal to that most distal to the origin of chromosomal replication. From Fig 2B and 2C it was revealed that, from the triple Chi array up to 15kb away from the DSB site, HJs were readily detected. In the next two fragments (Fig 2C), their accumulation gave much weaker signals. Accordingly, the quantification revealed that on the origin-proximal side of the DSB, the two fragments located between the triple Chi array and 15 kb from the DSB locus show the highest accumulation of HJs (between 4% and 5%) while the next fragment located between 18 kb and 24 kb from the DSB site only had around 1% of the probed DNA in the X-spike. The fragment farthest away did not show any additional HJ accumulation compared to the same strain which was not subjected to DSBs. Likewise, on the origin-distal side, the first two fragments from the triple Chi arrays contain the highest level of accumulation of HJs (between 2% and 3%). The proportion of HJs in the next fragment was smaller (less than 1%) and it was negligible in the 24–30 kb fragment. Interestingly, the two sides of the break did not accumulate similar amount of HJs. HJs accumulated 1.8 times more frequently on the origin-proximal side than on the origin-distal side suggesting that the two sides of the DSB might be processed differently despite the fact that the number and position of the Chi sites were identical in the first 15 kb on both ends of the break. More surprisingly, HJs were also easily detected in the central DSB fragment, which is devoid of Chi sites. Since the DSB repair pathway is not initiated unless RecBCD encounters a Chi sequence, we had expected no accumulation of HJs in the central 6 kb DSB fragment located between the two triple Chi arrays. However, more than 2% of the probed DNA from a strain subjected to DSBs was detected in the X-spike whereas only 0.68% of X-spike was detected in the control experiment. This confirms that HJs accumulated in the central DSB fragment, contrary to our expectation.

### Accumulation of HJs in the central DSB fragment

**Fig 3:**
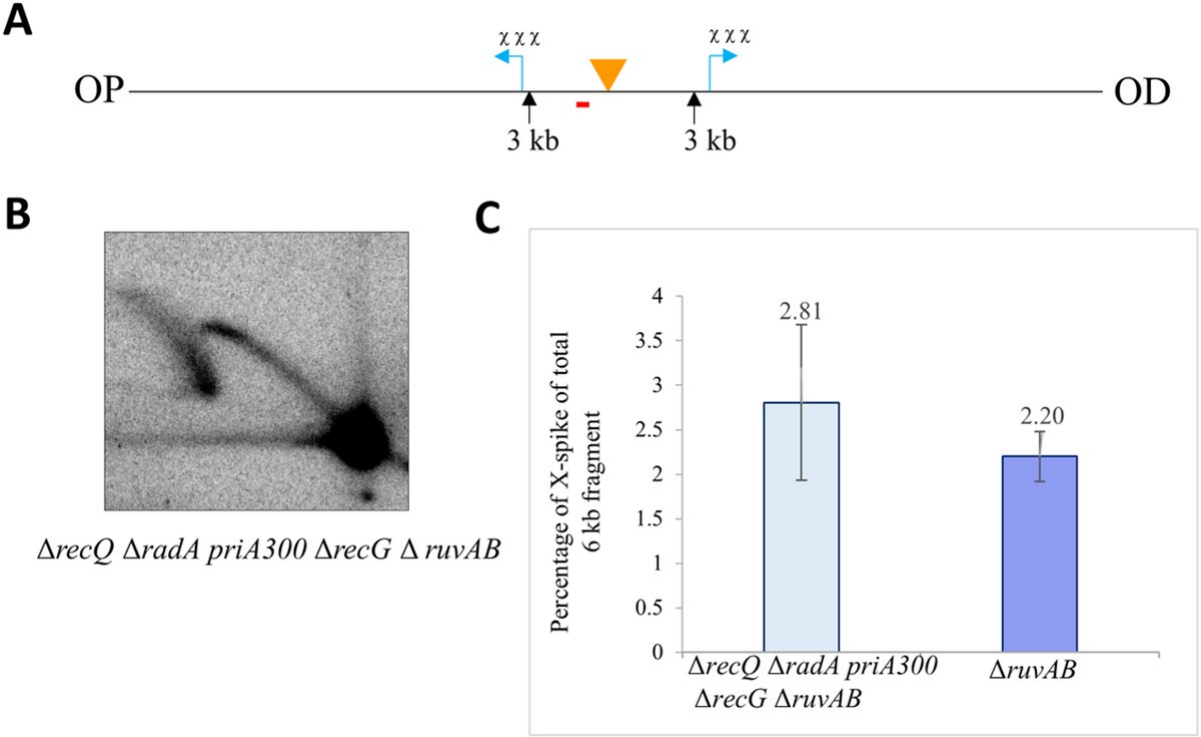
2-D gel analyses of the central DSB fragment in a Δ*recQ* Δ*radA priA300* Δ*recG* Δ*ruvAB* mutant strain and in a single Δ*ruvAB* mutant strain grown in presence of arabinose. (A) NotI digestion map of the *E*. *coli* chromosome between the triple Chi arrays in the mutant strain. The location of the palindrome is shown by an orange triangle and the Chi sites are indicated by blue arrows. NotI recognition sites of interest and their distance from the palindrome are marked by black arrows and numbers (in kb), respectively. The *lacZ* probe that was used to detect the 6kb fragment is marked by a red line placed below the restriction fragment. OP and OD mean origin-proximal and origin-distal side. (B) Southern blot of a native-native 2-D agarose gel of the crosslinked DNA fragment isolated from a bacterial cell culture grown in presence of arabinose for 60 minutes. The experiment was repeated three times independently. The strain used was DL7667 (Δ*recQ*, Δ*radA*, *priA300*, Δ*recG* and Δ*ruvAB*, *lacZ*::246) (C) Quantification of the HJs generated in crosslinked DNA samples isolated from a Δ*recQ* Δ*radA priA300* Δ*recG* Δ*ruvAB* mutant strain and from a single Δ*ruvAB* mutant strain, represented as the percentage of X-spike out of the total 6kb DNA fragment. Error bars represent the standard error of the mean where n = 3.

Surprisingly, HJs accumulated in the central DSB fragment in the absence of the branch migration activity of the RuvABC protein (Fig 2B and 2C). Since this central DSB fragment is devoid of any Chi site (Figs 1C and 3A), strand invasion and eventual HJ formation could not have taken place within this region. Either HJs are moving back by spontaneous branch migration or they are catalytically driven by some enzyme (or enzymes) other than RuvAB.

In order to investigate whether some other helicase was migrating the HJs back into the central fragment, several genes encoding helicases known to branch migrate joint molecules were deleted or disrupted in this Δ*ruvAB* mutant background. Genes encoding RecQ, RadA and RecG proteins were deleted, and the helicase activity of the PriA protein was inactivated by the *priA300* mutation [30]. The strain harbouring all these mutations was only modestly slower growing under the condition used (40min generation time in LB glucose at 37°C) compared to the Δ*ruvAB* single mutant (32min generation time in LB glucose at 37°C). It was likely that its viability benefitted from the known suppression of *recG* phenotypes by the *priA300* mutation. It was found that the percentage of DNA in the X-spike out of the total hybridised DNA from the central DNA fragment did not reduce in the strain with multiple deleted helicase activities compared to the single Δ*ruvAB* mutant strain (Fig 3B and 3C). This observation implies that HJs can move into the central DSB repair region between the triple Chi arrays in the absence of RuvAB and all of the above-mentioned helicase activities (RecG, RecQ, RadA and PriA).

### Correlation between RecA loading and the distribution of Holliday junctions

**Fig 4:**
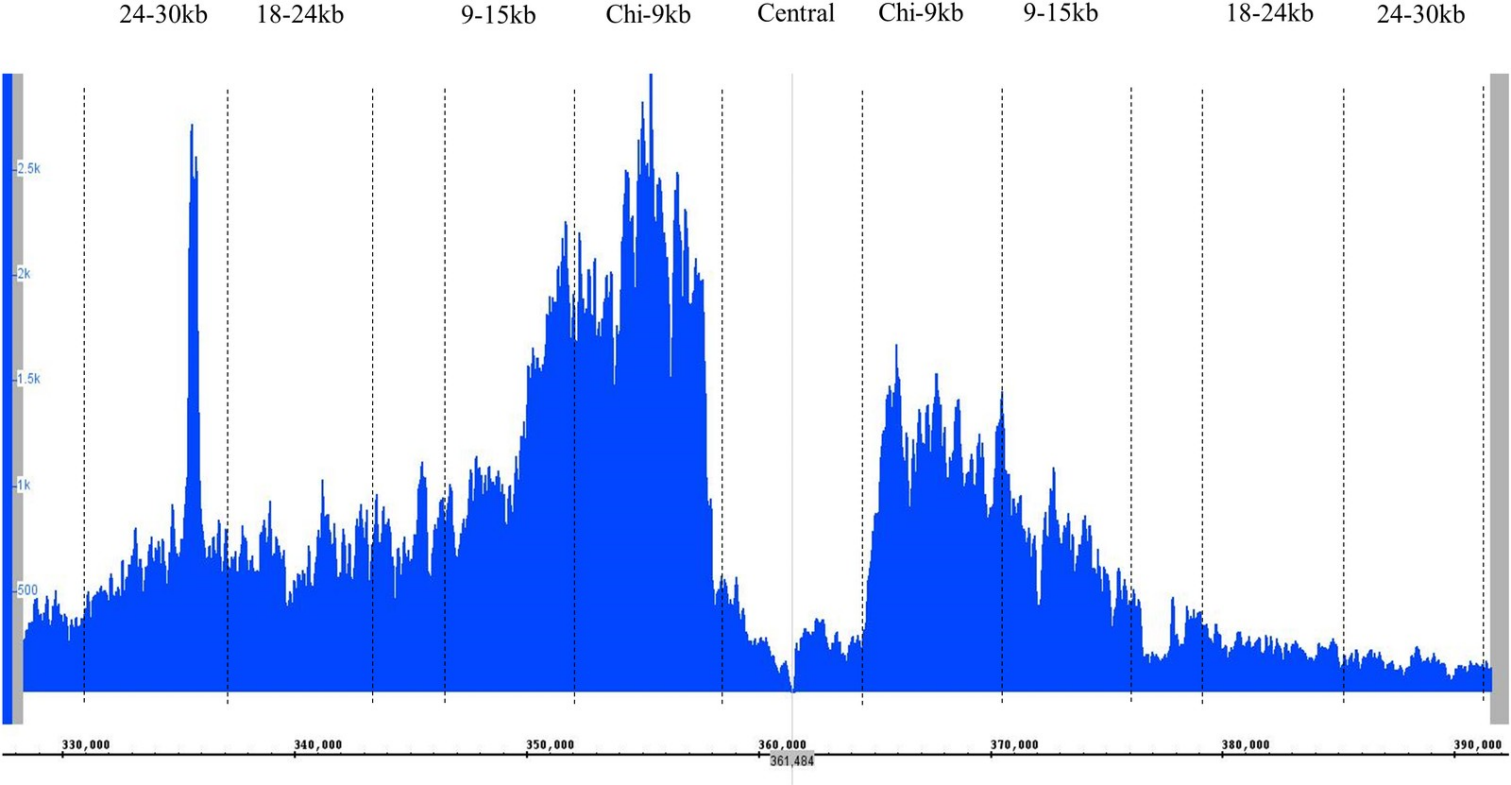
ChIP-Seq analysis on RecA loading in the 60kb DSB repairing region. ChIP-Seq analysis of DSB-dependent RecA loading in the *lacZ* region of strain DL5216 (*lacZ*::246bp palindrome, *mhpA*::3xChi, *lacZY*::3xChi) undergoing DSB repair. The 246 bp palindrome is located at 361268–361514 kb (marked by vertical grey line) on the map. The number of hits is depicted in blue. The NotI cutting sites are shown by black dashed lines and the resulting fragments are denoted at the top by their distances from the DSB site.

The RecBCD mediated HR pathway initiates with RecBCD action and the outcome of RecBCD action is manifested through the loading of RecA on DNA in a Chi-dependent manner. Therefore, chromatin immunoprecipitation combined with next-generation sequencing (ChIP-Seq) was performed to confirm the genome-wide localisation and quantification of RecA binding to DNA in response to this site-specific DSB repair event. This would allow us to correlate and compare two different steps of the pathway: RecA loading and HJ formation. Fig 4 shows the RecA ChIP-Seq analysis of the *lacZ* region of *E*. *coli* RuvAB^+^ cells undergoing DSBR. The regions of RecA enrichment are indicated by the peaks in the blue line and the positions where NotI cleavage sites have been inserted in the strains used for 2-D gel analysis are marked on the plot. It can be clearly seen that RecA loading and the distribution of HJs follow similar patterns, with the DNA in the Chi-9kb and 9-15kb fragments on both sides of the DSB site displaying greater RecA enrichment and higher HJ accumulation than the outer two fragments. Since the triple Chi arrays are located in the Chi-9kb fragments, these observations are in accordance with the *in vitro* biochemistry of RecBCD enzyme, notably that following a DSB, RecA protein is loaded onto DNA in a Chi-dependent manner [31]. It can be seen that RecA enrichment is higher in the Chi-9kb fragments than in the 9-15kb fragments (on both sides of the DSB), while HJ accumulation is very similar in these fragments. These observations are also expected as HJs are predicted to form following RecA loading and strand invasion. Therefore, their accumulation is expected to be further away from the DSB site. Furthermore, the absence of any RecA enrichment between the triple Chi arrays indicates that RecBCD has not started its recombinogenic function in this region and therefore supports our hypothesis that HJs detected in the central fragment between the Chi sites must have moved back towards the DSB site.

### Detection of 3-way DNA structures across the DSB region in Δ*ruvAB* mutants

**Fig 5:**
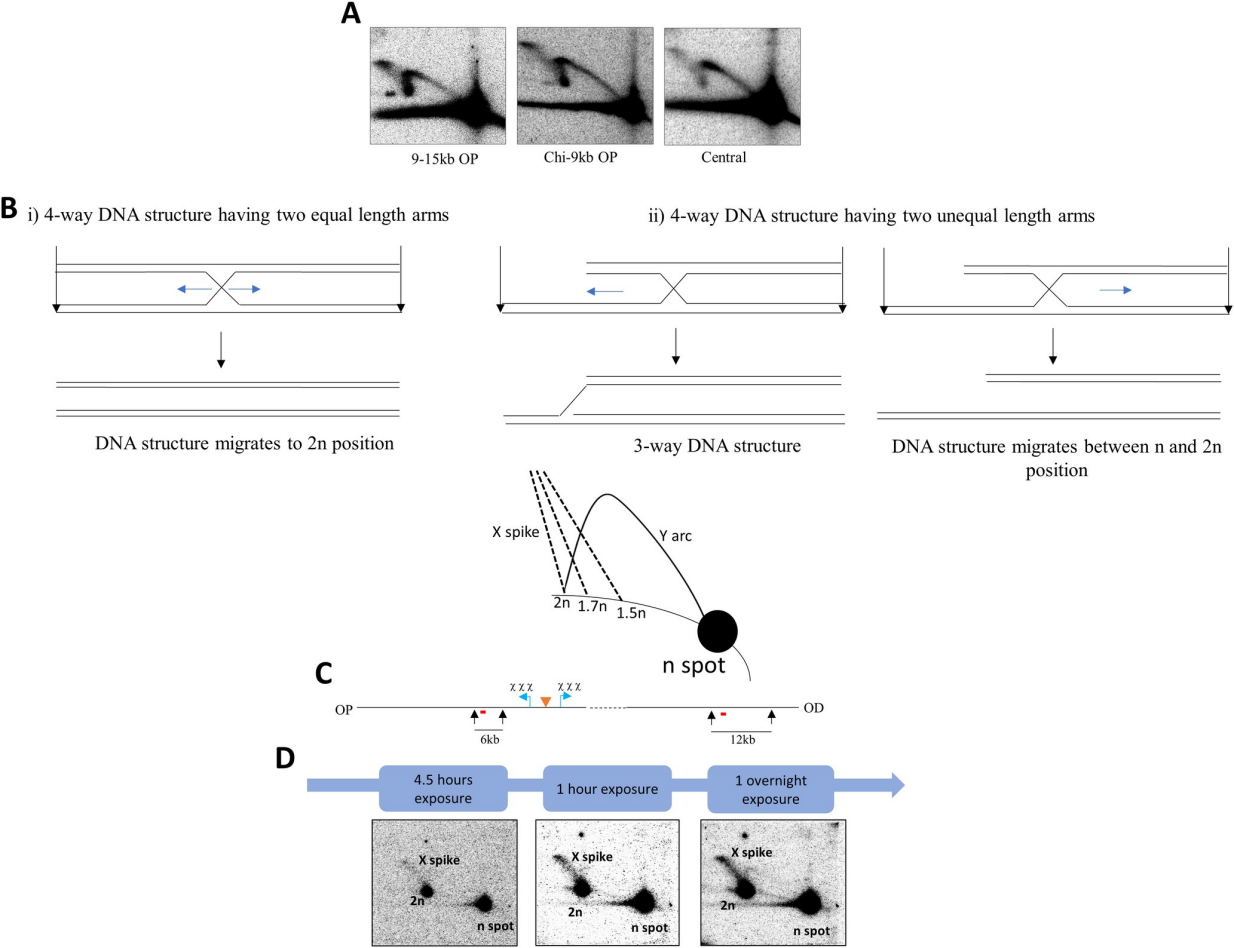
2-D gels of non-crosslinked DNA molecules and determination of the nature of the branched DNA structures accumulated along the X-spike that is detected in the Southern blot of 2-D gel electrophoresis of crosslinked DNA molecules from a Δ*ruvAB* strain. A) 2-D gels of non-crosslinked 9–15 kb OP, Chi-9 kb OP and central fragments for Δ*ruvAB* strains (DL7259, DL7253 and DL7272, respectively) containing the palindrome, grown in the presence of 0.2% arabinose for 60 minutes. B) Schematic representation of the predictions about the emplacement where the X-spike reaches the linear arc according to the two different hypotheses C) NotI digestion map of the *E*. *coli* chromosome in which the location of the palindrome is shown by an orange triangle and the Chi sites are indicated by blue arrows. NotI recognition sites of interest and the fragments resulting from NotI digestion are marked by black arrows and numbers (in kb), respectively. The *prpD* and *ydeA* probes that were used to detect the 6kb and 12kb fragments are marked by red lines placed below the restriction fragments. OP and OD mean origin-proximal and origin-distal sides. D) Detection of the n spot using *prpD* probe that recognises the 6kb *prpD* fragment and the 2n spot using *ydeA* probe that recognises a 12 kb fragment approximately 1.2Mb away from the palindrome. The fragments resulted from NotI digestion of crosslinked DNA isolated from bacterial cell cultures grown in LB medium supplemented with 0.2% arabinose for 60 minutes. With increasing film exposure time, both linear and branched DNA species become detectable. Therefore, after 4.5 hours of exposure, the blot starts to show the 6kb linear and 12kb branched fragments as detected by the *prpD* probe and the 12kb linear fragment as detected by the *ydeA* probe. After overnight exposure the 12 kb branched fragment becomes strongly detectable. The strain used was DL7577 (Δ*ruvAB lacZ*::246).

In order to determine what happens to the branched structures when they are not stabilised by crosslinking, non-crosslinked DNA molecules were also analysed. 2-D gel analysis revealed the accumulation of 3-way DNA structures across the DSB region (Figs 5A and S2). These structures were less clearly visible in the crosslinked samples where HJs were more prominent (Figs 2B and S1). Although HJs could be observed in the non-crosslinked DNA fragments, part of the X-spikes was always covered by the Y-arcs thereby leading us to conclude that quantifying the HJs from non-crosslinked DNA analysis would give us an inaccurate estimation of their accumulation. Fig 5A shows examples of Southern blots of non-crosslinked DNA for the central fragment and the immediate next two fragments that are located between the Chi array and 15kb away from the palindrome on the origin-proximal side. Representative examples of Southern blots of the rest of the fragments are shown in S2 Fig.

### 3-way DNA structures detected in DSB repair do not derive from *in vitro* branch migration of HJs

We further investigated the reason for the observed differences between the structures of the branched intermediates that accumulated in the presence and absence of DNA crosslinking. Since the strains and the growth conditions of the cultures were identical for both crosslinked and non-crosslinked DNA samples, two hypotheses potentially explaining the difference were envisaged. In the first hypothesis, in the absence of crosslinking, HJs would not be stable and would potentially be converted into 3-way DNA structures by branch migration, represented by a Y-arc. In the second hypothesis, the X-spike and the Y-arc consist of different DNA species. When the DNA is not crosslinked, HJs fall apart into linear molecules, leaving the more stable 3-way structures in the Y-arc, the visualisation of this Y-arc being enhanced by the absence of DNA crosslinks that interfere with hybridisation in Southern blots.

According to the first hypothesis, if 4-way HJs can be converted to 3-way structures by *in vitro* branch migration, they must consist of dsDNA molecules with unequal length arms, and the lower part of the X-spike in the crosslinked DNA might therefore be expected to reach the linear arc somewhere between the n spot and 2n spot representing the un-replicated and replicated DNA, respectively (Fig 5B). On the other hand, if the HJs consist of two equal full-length dsDNA molecules, as predicted by the second hypothesis, they will fall apart into two linear molecules in the absence of crosslinks and as a result they will be detected as part of the n spot in the autoradiograph. In this case, the X-spike in the crosslinked DNA analysis should reach the linear arc at approximately the 2n location, which represents the (nearly) fully replicated linear DNA fragment. Therefore, the nature of the 4-way structures contained within the X-spike was investigated to determine which of these hypotheses is most probable. The predictions are explained in Fig 5B. In order to find out where the X-spike reaches the linear arc relative to the n and 2n locations, these positions on the linear arc needed to be determined. For this purpose, a Southern blot of the 2-D gel analysis of one of the NotI digested fragments which is 9 to 15kb away from the palindrome on the origin-proximal side, was used. The blot was hybridised with two different radioactive probes sequentially. The first one was used to detect both the linear 6kb fragment as the n spot and the 4-way DNA structures accumulated within this 6kb fragment as the X-spike, and the second one was used to recognise a 12kb fragment, located somewhere else on the chromosome, thereby detecting the 2n spot on the linear arc as this could not be done with the first probe. After a short exposure time (1 hour), the autoradiograph showed two intense spots on the linear arc representing the linear 6kb and 12kb fragments and a very faint X-spike (Fig 5D). However, with increasing exposure time, a stronger X-spike was visible which reached the linear arc exactly at the 2n spot, implying that the 4-way DNA structures contained within the X-spike are joint DNA molecules that have two equal full-length dsDNA molecules. These observations coupled to a consistently 2–3 fold lower signal across entire blots following DNA crosslinking favour the second hypothesis, that these 4-way and 3-way DNA structures were of different origin.

### 3-way DNA structures detected in DSB repair are not D-loops

Once it was established that the 3-ways structures observed in the non-crosslinked DNA analysis did not originate from molecules detected as 4-way structures in the crosslinked DNA, the possible origins of the structures contained within the Y-arc were further explored. We considered the possibilities that these Y-arcs represent either D-loops or replication forks. The observation that D-loops can dissociate by branch migration, facilitated by high temperature and low salt concentrations [32], was used to distinguish between these possibilities. After NotI digestion of non-crosslinked DNA, the high salt restriction enzyme buffer was removed from the agarose plugs by washing in TE buffer. The plugs were then incubated at 37°C from a few hours to overnight or at 45°C for a few hours to permit dissociation of D-loops. When the DNA was analysed by 2-D gel electrophoresis alongside plugs that had been digested and treated using the usual method (incubation in the restriction enzyme buffer containing high salt), similar yields of Y-arcs were detected irrespective of the plug treatment (S3 Fig). These observations indicate that the DNA species in the Y-arcs did not behave as expected for D-loops and therefore most likely represent replication forks. Since these structures were largely detected in the samples where a DSB was induced (Fig 5A), they must be associated with the presence of the DSB.

### 3-way DNA structures detected in DSB repair do not derive from chromosomal DNA replication

Having established that the Y-arcs most likely represent replication forks, formed as a consequence of DSB induction, we set out to test whether they originate from DSB repair or represent chromosomal replication forks trapped by unresolved repair intermediates such as HJs. The DSB occurs at the palindrome following the passage of a replication fork through the sequence. Therefore, we hypothesised that if DNA replication forks originating from the chromosomal origin were being trapped at unresolved branched intermediates, it would take some time for these subsequent replication forks to arrive at the DSB region following induction of DSB repair. More time would provide more opportunity for such forks to become trapped. Therefore, the growth rate of the cells was decreased so that the quantity of Y-arcs, as a function of the number of replication cycles, could be more easily determined. By growing the cell culture in M9 minimal medium supplemented with 0.5% glucose the doubling time was increased to approximately 60 minutes, compared to a generation time of approximately 32 minutes in LB glucose medium. For a given cell that had experienced a palindrome/SbcCD-dependent break, the arrival of the next replication fork at the DSB site must take at least one generation time. This is due to the fact that palindrome/SbcCD-dependent breaks are replication dependent and the time between subsequent replication firings of the origin equals the generation time [33]. During a second generation time, all breaks caused by passage of former replication forks initiating DSB repair will have the opportunity to trap subsequent replication forks. So, we predicted that no trapped chromosomal replication forks would be detectable until one generation time following DSB induction and the number of such trapped forks would increase through a second generation time.

It was found that both 4-way and 3-way structures were accumulated in cell cultures when grown for less than one generation time in the M9 medium following induction of SbcCD (0.8 generation time) (S1 Table). When the culture was grown for more than one generation time following induction of SbcCD (1.6 generations time), more 4-way structures were detected. However interestingly the percentage of 3-way structures remained similar. The 4-way/3-way structures ratio increased when the culture was grown for 1.6 generation times compared to the culture grown for 0.8 of a generation time (S1 Table). These observations exclude the possibility that the 3-way structures detected derive from trapping of chromosomal replication forks that have run into unresolved HJs. We conclude therefore that the 3-way structures detected in non-crosslinked DNA analyses most likely represent repair forks implicated in DNA synthesis to restore the DNA lost during DSB repair.

### The distribution of repair forks mirrors the distribution of HJs

**Fig 6:**
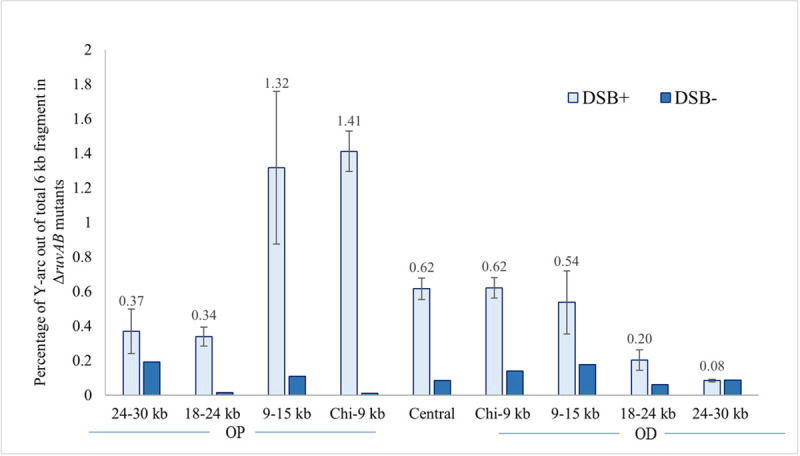
Distribution of 3-way DNA structures across the *lacZ* region of Δ*ruvAB* mutant strains as a function of induction of DSB repair. Quantification of the signal intensities of 3-way DNA structures generated across the 60kb region following a DSB. This analysis was carried out on non-crosslinked DNA samples and 3-way DNA structures were quantified as the percentage of Y-arc out of the total 6kb DNA fragment (DSB^+^). The quantification of Y-arcs from control blots (2-D gel analysis of non-crosslinked DNA fragments isolated from bacterial cell culture grown in presence of glucose) is also shown (DSB-). The strains used were DL7271 (Δ*ruvAB* 24-30kb OP), DL7270 (Δ*ruvAB* 18-24kb OP), DL7259 (Δ*ruvAB* 9-15kb OP), DL7253(Δ*ruvAB* Chi-9kb OP), DL7272 (Δ*ruvAB* Chi-Chi), DL7251 (Δ*ruvAB* Chi-9kb OD), DL7258 (Δ*ruvAB* 9-15kb OD), DL7261 (Δ*ruvAB* 18-24kb OD) and DL7262 (Δ*ruvAB* 24-30kb OD). OP and OD represent origin-proximal and origin-distal sides. Error bars represent the standard error of the mean where the number of replicates for DSB^+^ strains is 3 and DSB^-^ strains is 1.

The distribution of the 3-way DNA structures across the DSB repairing region was determined from the non-crosslinked DNA analyses. The result is shown in Fig 6 as represented by the percentage of the amount of DNA in the Y-arc out of the total probed DNA. As can be seen, the origin-proximal and origin-distal sides showed different accumulations of Y-arcs, with a higher proportion of the branched structures on the origin-proximal side (approximately 2.4 times more) compared to the origin-distal side. While, on the origin-proximal side, approximately 1.5% of the probed DNA was detected along the Y-arc in the Chi-9kb and 9-15kb fragments away from the palindrome, on the origin-distal side, less than 1% of the probed DNA in those two fragments could be detected in the Y-arc. Importantly, the central DSB fragment also showed accumulation of 3-way DNA structures within the region. Having established that the 3-way junctions most likely represent repair forks, it is interesting to note that their distribution closely reflects the distribution of HJs (Fig 2C).

### Role of exonuclease I in DSB repair

**Fig 7:**
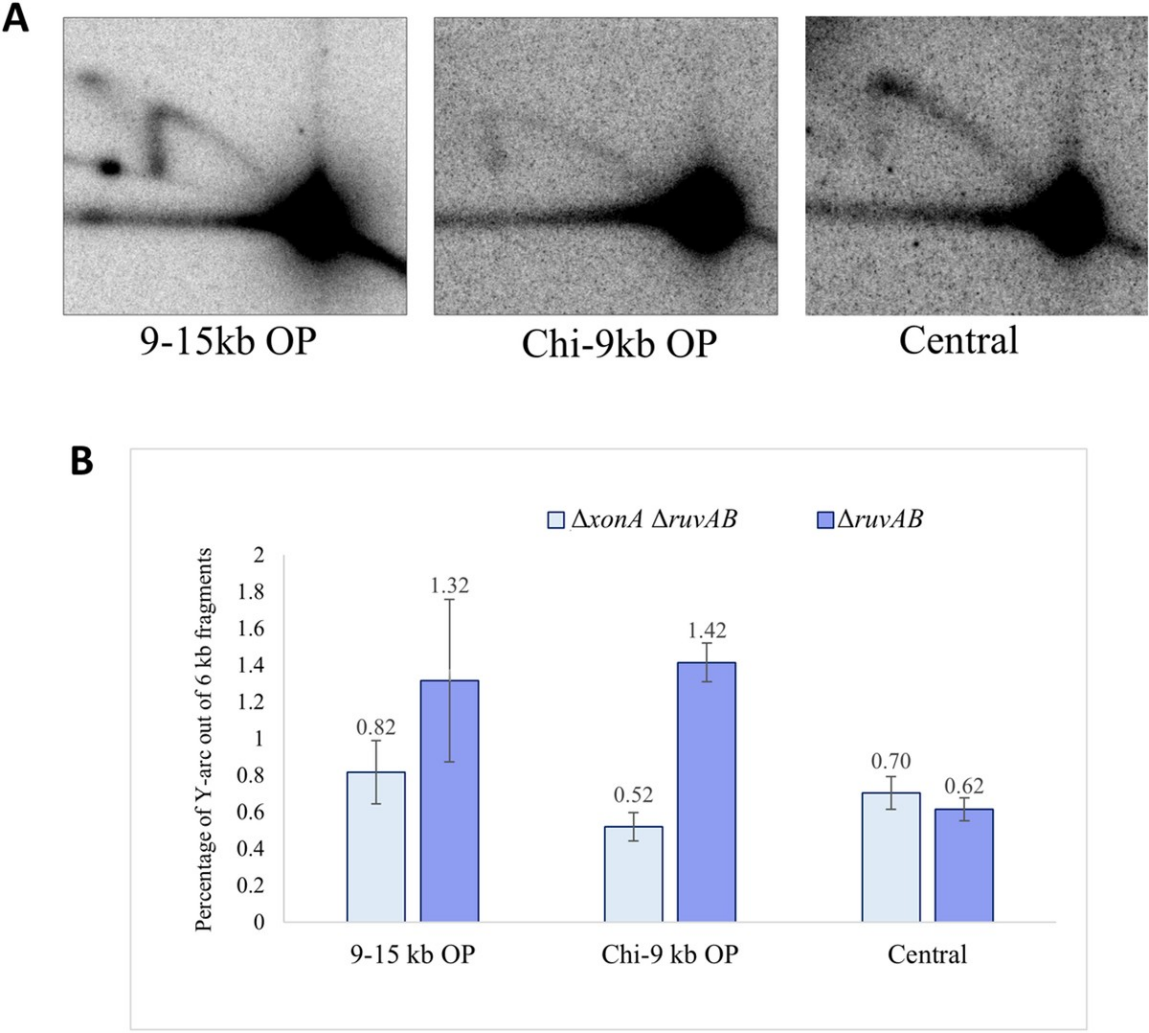
Distribution of repair forks across the central fragment, the Chi-9kb fragment and 9-15kb fragments on the origin-proximal side of the DSB isolated from Δ*ruvAB* single mutant and Δ*xonA* Δ*ruvAB* double mutant strains grown in presence of arabinose. A) 2-D gels of the non-crosslinked 9-15kb OP, Chi-9kb OP and central fragments for Δ*xonA* Δ*ruvAB* strains containing the palindrome, grown in the presence of 0.2% arabinose for 60 minutes. B) Quantification of the repair forks generated in the non-crosslinked DNA samples represented as the percentage of DNA in the Y-arc out of the total 6kb DNA fragments. OP and OD mean origin-proximal and origin-distal sides. Error bars represent the standard error of the mean where n = 3. The strains used were DL7840 (Δ*xonA* Δ*ruvAB* 9-15kb OP), DL7839 (Δ*xonA* Δ*ruvAB* Chi-9kb OP), DL7841 (Δ*xonA* Δ*ruvAB* Chi-Chi), DL7259 (Δ*ruvAB*, 9-15kb OD), DL7253 (Δ*ruvAB*, Chi-9kb OD) and DL7272 (Δ*ruvAB*, Chi-Chi).

Given that DNA repair forks and HJs could form efficiently up to 12kb away from a Chi array implicated in initiating recombination via interaction with RecBCD, we suspected that other nucleases might play parts in defining the location of strand-invasion leading to the formation of repair forks. We hypothesised that a candidate for enabling strand-invasion away from the Chi site was exonuclease I (ExoI), the product of the *xonA* gene. This exonuclease degrades single-stranded DNA in a 3’-5’ direction and so might displace a hypothetical invading 3’ single-strand end away from the Chi site where the 3’-5’ nuclease activity of RecBCD has arrested. To investigate the role of exonuclease I in DSB repair, three Δ*xonA* Δ*ruvAB* double mutant strains, in which NotI digestion would enable analysis of the central fragment between the Chi arrays, the Chi-9kb fragment and the 9-15kb fragment on the origin-proximal side of the DSB were constructed and analysed (Fig 7A) as these are the fragments where the Y-arc could be quantified with confidence. In the absence of ExoI, we predicted an increase in the quantity of Y-arc in the Chi-9kb fragment and a decrease in the 9-15kb fragment since the 3′ ssDNA overhang generated by RecBCD would not be degraded, and as a result invasion might occur closer to the Chi array. However, the percentage of Y-arc was reduced in both Chi-9kb and 9-15kb fragments in the Δ*xonA* derivative of the Δ*ruvAB* mutant, which suggested that digestion of a 3’-ended single-strand by ExoI stimulates initiation of repair synthesis in both DNA fragments. The results obtained from the 2-D gel analyses on these three non-crosslinked 6kb fragments are shown in Fig 7B. The 2-D gel quantification for the corresponding crosslinked DNA fragments is shown in S4 Fig that shows a similar reduction in the percentage of the HJs in the Chi-9kb and 9-15kb origin-proximal fragments.

### Role of RecJQ in DSB repair

**Fig 8:**
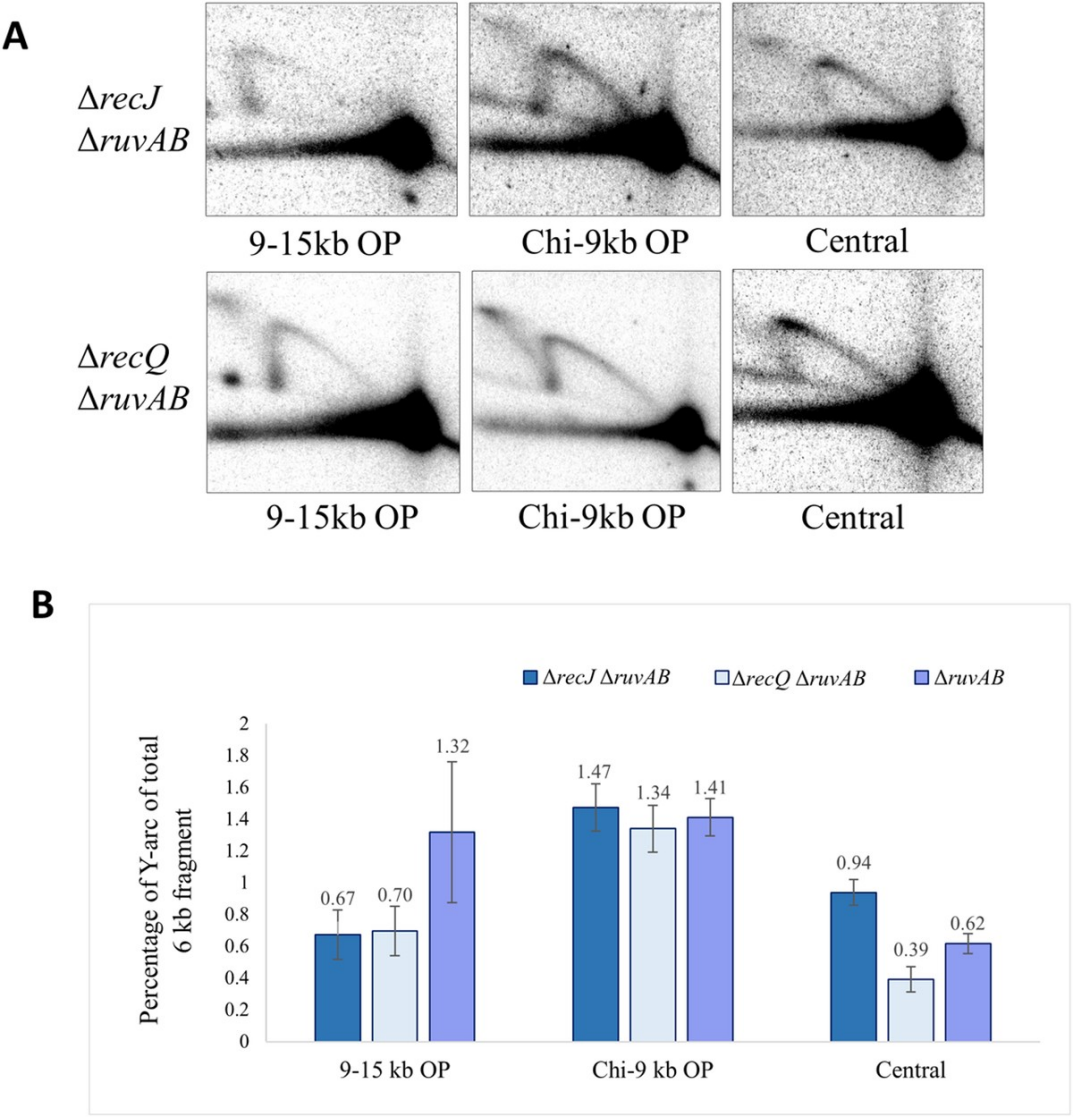
Distribution of repair forks across the central, Chi-9kb and 9-15kb fragments on the origin-proximal side of the DSB isolated from Δ*ruvAB* single mutant, Δ*recJ* Δ*ruvAB and* Δ*recQ* Δ*ruvAB* double mutant strains grown in presence of arabinose. A) 2-D gels of the non-crosslinked 9-15kb OP, Chi-9kb OP and central fragments for Δ*recJ* Δ*ruvAB* and Δ*recQ* Δ*ruvAB* strains containing the palindrome, grown in the presence of 0.2% arabinose for 60 minutes. B) Quantification of repair forks generated in the non-crosslinked DNA samples represented as percentage of DNA in the Y-arc out of the total 6kb DNA fragments. OP and OD mean origin-proximal and origin-distal sides. Error bars represent the standard error of the mean where n = 3. The strains used were DL7859 (Δ*recJ* Δ*ruvAB*, 9-15kb OP), DL7857 (Δ*recJ* Δ*ruvAB*, Chi-9kb OP), DL7827 (Δ*recJ* Δ*ruvAB*, Chi-Chi), DL7591 (Δ*recQ* Δ*ruvAB*, 9-15kb OP), DL7874 (Δ*recQ* Δ*ruvAB*, Chi-9kb OP), DL7588 (Δ*recQ* Δ*ruvAB*, Chi-Chi), DL7259 (Δ*ruvAB*, 9-15kb OP), DL7253 (Δ*ruvAB*, Chi-9kb OP) and DL7272 (Δ*ruvAB*, Chi-Chi).

The unexpected observation that repair forks are not elevated near Chi in absence of ExoI, led us to investigate the role of the 5’ to 3’ exonuclease RecJ, as we realised our understanding of the roles of accessory nucleases was incomplete. Three Δ*recJ* Δ*ruvAB* double mutant strains in which NotI digestion would result in the same three fragments, the central one and the two fragments on the origin-proximal side of the DSB, were analysed. Our initial prediction was that absence of RecJ might have little effect, given our expectation that strand invasion was mediated by a 3’ single-stranded DNA end, generated by RecBCD, that would be unaffected by the presence or absence of a 5’-3’ exonuclease such as RecJ. However, the percentage of Y-arc was found to be reduced in the 9-15kb fragment compared to the Chi-9kb fragment (Fig 8). This observation suggests that when RecJ degrades 5′ ssDNA, a longer 3′ ssDNA overhang can be generated which increases the possibility of invasion further away from the triple Chi array and therefore repair forks tend to get accumulated and detected in higher amounts in the 9-15kb fragment. It is known that, in *E*. *coli*, although RecJ nuclease alone is capable of digesting DNA with a 5´ ssDNA overhang [22], RecJ cannot resect duplex DNA that is either blunt-ended or terminated with 3′ ssDNA, unless such DNA is unwound by a helicase such as RecQ [34]. Therefore, we further investigated whether RecJ acts on its own or in conjunction with RecQ. Quantification of the accumulation of Y-arcs in the Chi-9kb and 9-15kb fragments in the absence of RecQ revealed that the Δ*recQ* mutation had a similar effect to Δ*recJ* mutation on repair fork distribution. The quantification of the 2-D gel analyses of the non-crosslinked DNA from these mutant strains is shown in Fig 8 along with Δ*ruvAB* mutant strains. The quantification of the 2-D gel analyses of the crosslinked DNA from these mutant strains is shown in S5 Fig. Here it can be seen that HJs are reduced in both the 9-15kb and Chi-9kb origin-proximal fragments again suggesting a role for RecJQ in stimulating recombination by processing the 5′-ended DNA strand following the action of RecBCD at Chi.

## Discussion

Analysis of 2-D gels has revealed the distribution of recombination intermediates accumulated in a Δ*ruvAB* mutant in the *lacZ* region of the *E*. *coli* chromosome following induction of a repairable, replication-dependent DNA double-strand break (DSB). The Δ*ruvAB* mutant strains used were devoid of all endogenous Chi sites within a 15-kb region on either side of the break, leaving only synthetic triple-Chi arrays introduced 3kb either side of palindrome. Both intra-strand crosslinked, and non-crosslinked DNA samples were analysed allowing us to visualise both Holliday junctions (HJs) and repair forks.

### HJ formation in RecBCD-mediated DSB repair occurs mainly over a wide region of 12kb of DNA from the recombination initiating Chi site

One key objective of investigating the distribution of HJs following a site-specific DSB *in vivo* was to shed light on how far DNA ends are processed before strand invasion occurs, as these events are difficult to investigate due to their transient nature. On both origin-proximal and origin-distal sides, HJs were observed to accumulate as X-spikes mainly up to 12kb away from the Chi array responsible for initiating the majority of recombination events and 15kb away from the DSB site (Fig 2C). After the Chi array, on the origin-proximal side, there are 4 additional endogenous Chi sites in the first 50kb region. Therefore, it is difficult to be certain whether an accumulation of HJs on the origin-proximal side results from recombination events that initiated at the triple Chi array or from the movement of HJs that were generated at further Chi sequences and travelled back towards the DSB. Nevertheless, the distribution of RecA protein (Fig 4) suggests that the majority of recombination events have indeed been initiated at the synthetic Chi array. On the origin-distal side, there are no endogenous Chi sites for 100kb after the synthetic Chi array and no significant accumulation of HJs beyond the 12kb fragment from that array (Fig 2C). The distributions of RecA and of HJs observed suggest that, in the population of recombining cells, there is a wide distribution of strand-invasion events leading to HJs forming over an extended region from close to Chi to 12kb away from Chi.

The HJ distribution revealed approximately two-fold more HJs on the origin-proximal side of the DSB compared to the origin-distal side, and similar observations were made about the distribution of RecA. This has been seen previously and has been explained by RecBCD catching up with the ongoing replication fork before it reaches the triple Chi array on the origin-distal side converting a two-ended break to a one-ended break in approximately 50% of events [16] (Fig 1B).

### Repair DNA synthesis can occur prior to the resolution of HJs and may cause HJ relocation

The distribution of HJs was consistent with the distribution of RecA protein, apart from in the central 6kb fragment where HJs can be detected despite no RecA binding. The absence of RecA binding is expected from the known activity of RecBCD enzyme, which can only load RecA following interaction with Chi [31]. However, the presence of HJs in this region is unexpected and suggests their ability to branch migrate towards the DSB despite the absence of RuvAB activity. Since the HJs migrate in 2-D gels as expected for full-length dsDNA molecules, any DNA that had been lost by RecBCD processing between the DSB site and Chi must have been restored. This implies that repair DNA synthesis, that is known to accompany the RecBCD pathway, can take place before HJs are resolved. In order to test the nature of the activity responsible for the migration of these HJs, a mutant strain was investigated in which five known recombination helicase activities were absent. However, the movement of HJs toward the central fragment still took place in the absence of the helicase activities of RecG, RecQ, RadA and PriA in addition to the absence of RuvAB.

One possible explanation could be that subsequent replication forks originating from the chromosomal origin push HJs towards the DSB site. However, replication from the origin would push all structures towards the terminus whereas the origin-proximal and origin-distal HJ distributions looked similar. An attractive alternative possibility is that repair forks moving towards the DSB site play an indirect role in pulling HJs towards the central region. Repair forks are expected to run in front of their respective HJs, accumulating positive supercoils ahead of themselves and negative supercoils behind them [35]. Therefore, a zone of negative supercoiling is predicted to lie between the repair forks and their HJs. The HJs will spontaneously move towards the region of negative supercoiling in front of them and in this way, repair forks may pull HJs towards the central DSB site.

### The 3-way junctions detected as Y-arcs in the absence of DNA crosslinking are likely to be repair forks

A striking difference was observed between crosslinked and non-crosslinked DNA samples in 2-D gel analyses. While in crosslinked DNA, mainly X-spikes representing 4-way DNA structures were detected, in the non-crosslinked DNA analyses primarily Y-arcs representing 3-way DNA structures were observed. We first determined that the X-spikes consist of two full length dsDNA molecules joined together through a junction, so it was not possible for these 4-way structures to convert into the observed 3-way structures by *in vitro* branch migration following their extraction from cells. We then determined that the 3-way structures observed were not sensitive *in vitro* to low salt conditions and moderate temperatures that we predicted would dissociate D-loops but not replication forks. Finally, by slowing down the growth rate of cells, we were able to determine that the 3-way structures observed were not consistent with origin-derived chromosomal replication forks being arrested by HJs formed at the site of DSB repair. We therefore consider that the 3-way junctions detected are most likely to be repair forks, required to restore DNA lost during the resection of DNA by recombination nucleases including RecBCD enzyme. The presence of repair forks amongst the intermediates of this DSB repair reaction is predicted by the requirement for PriA activity for cell viability following palindrome/SbcCD mediated DSB formation [12]. This explanation suggests that both 3-way and 4-way structures are present in the crosslinked samples. However, in the absence of crosslinking the 4-way structures are sensitive to *in vitro* branch migration and so are substantially lost in the non-crosslinked samples. On the other hand, the visibility of the 3-way structures in the non-crosslinked samples is enhanced because of a 2–3 fold stronger signal detected in the absence of DNA crosslinking, as crosslinking most likely interferes with transfer to the nylon membrane and hybridisation with the probe.

### Exonuclease I and RecJQ cooperate with RecBCD in DSB repair

Previous studies have shown that the functions of ExoI and RecJ nucleases contribute to RecBCD action. Early work showed that an *sbcB* mutant (defective for ExoI) limited the degradation of EcoK restricted bacteriophage lambda in the presence of RecBCD, but not in *recB* or *recBC* mutants, suggesting that exonuclease I has access to products of RecBCD action [36]. Degradation of bacteriophage T4 gene *2am*, which cannot protect itself from attack by RecBCD, is also reduced in a *xonA* mutant (also defective for ExoI) and degradation of the same phage in a *recD* mutant was reduced in both *xonA* and *recJ* mutants [37]. These genes have also been shown to affect RecBCD-mediated recombination. Transduction, requiring recombination in a short (3kb) region of homology was reduced in *sbcB* and *recJ* mutants in the presence of RecBCD or RecBC (*recD* mutant) [38]. These effects were much reduced when longer regions of homology were available, leading the authors to suggest that exonuclease I and RecJ promote a rate limiting step in RecBCD mediated recombination. Additionally, conjugational recombination in the presence of RecBCD is reduced slightly in a *xonA* mutant and is 10-fold reduced in a *xonA recJ* double mutant [39]. In bacteriophage lambda crosses, *sbcB* mutants affect the distribution of recombination events away from a Chi site acting opposite a DNA heterology [40]. A further study revealed that *xonA*, *recJ* and *xonA recJ* double mutants all depress the frequency of RecBCD-mediated recombination stimulated by Chi and *sbcB* and *xonA recJ xseA* mutants affect the distribution of recombination events away from a Chi site acting opposite a DNA heterology [41]. These observations are complex. However, together they point to accessory roles of exonuclease I and RecJ in RecBCD recombination stimulated by Chi.

**Fig 9:**
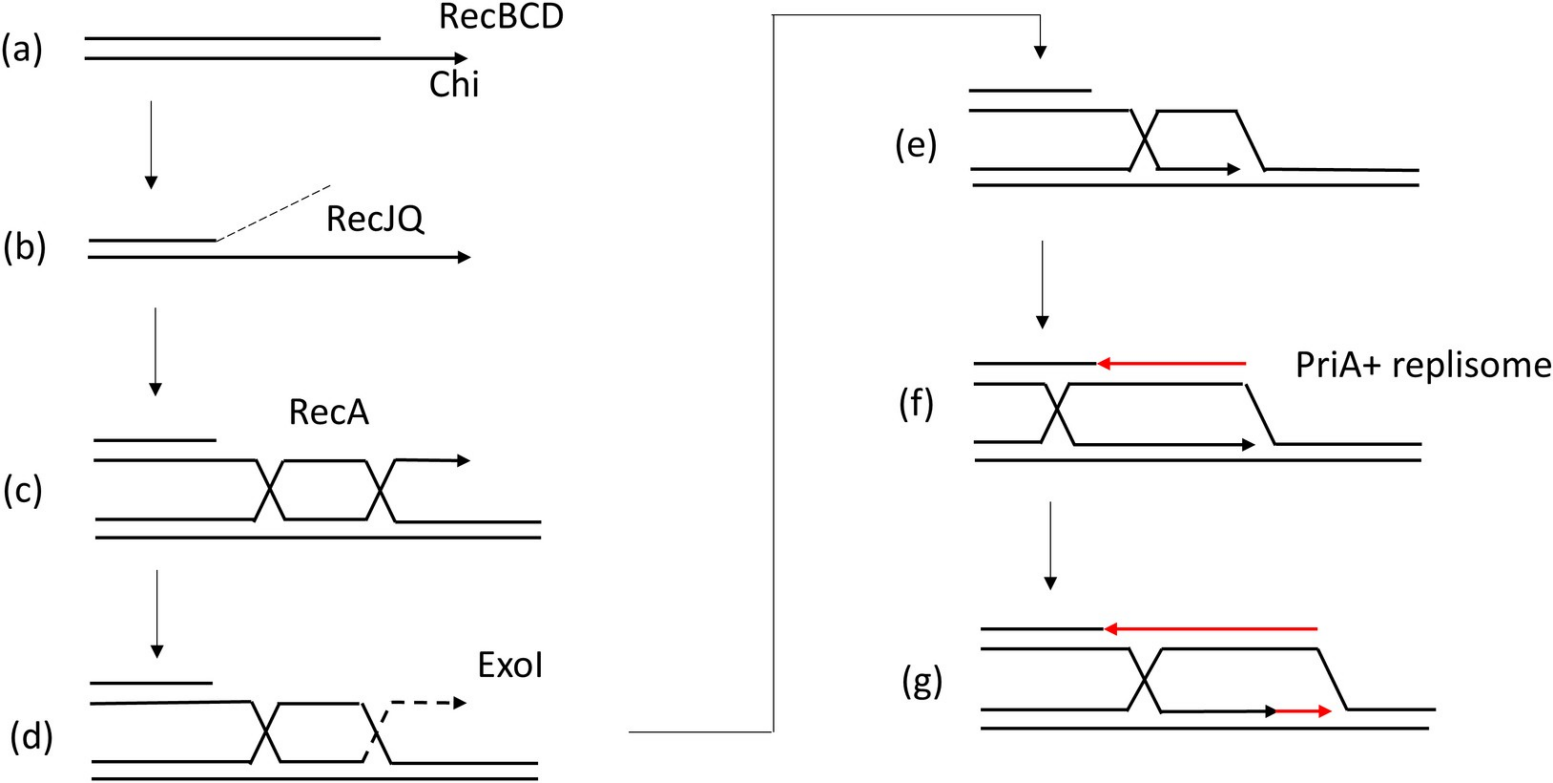
Model of formation of *E*. *coli* DSB repair intermediates. At a DSB, RecBCD resects the DNA end up to a Chi site, whereupon it continues to degrade the 5’ ended strand to produce a molecule with a 3’ single-stranded overhang (a). This molecule can be acted upon by the RecJ nuclease and RecQ helicase to extend the 3’single-stranded overhang (b). RecA binds to the single-strand overhang and initiates synapsis and strand exchange at a region of DNA homology. This creates a paranemic joint (c). ExoI digests the extruding 3’ tail (d) to generate a D-loop (e). PriA initiates the assembly of a replisome (f) and ensures that leading-strand synthesis is coupled to lagging-strand synthesis [48]. DNA replication towards the DSB site generates a wave of negative supercoiling behind the fork that (in the absence of RuvAB) drags the HJ towards the DSB site (g).

We show here that, in the absence of the 3′-5′ exonuclease ExoI, the quantity of repair forks showed a tendency to go down in both Chi-9kb and 9-15kb fragments on the origin-proximal side of the break. Since a 3′ overhang cannot be degraded by exonuclease I in a Δ*xonA* mutant, we had expected that invasion might take place closer to the Chi array and consequently the accumulation of Y-arc would increase in the Chi-9kb fragment and reduce in the 9-15kb fragment. However, the fact that Y-arc accumulation reduced in both fragments in a Δ*xonA* mutant, shows that ExoI stimulates the formation of repair forks irrespective of the distance from Chi. On the other hand, in the absence of the 5′-3′ exonuclease RecJ, the quantity of repair forks in the 9-15kb fragment was reduced compared to the condition when RecJ is present. A similar effect was observed in the absence of RecQ helicase, suggesting that RecJ and RecQ are working together. These observations can be explained in the following way (Fig 9): during RecBCD-mediated HR pathway, the 5′ end after being resected by RecBCD, may be further resected by the 5′ exonuclease activity of RecJQ, thus extending the 3′ ssDNA overhang. If invasion into a homologous DNA duplex then happens anywhere along the long DNA overhang, a paranemic junction will be the first joint molecule to form. The resulting D-loop will have a 3′ end that extrudes from the loop, as has been suggested for transformation in *Streptococcus pneumoniae*, and where RadA has been proposed to allow pairing of the 3’ end [42]. In *E*. *coli*, we propose that ExoI can perform this role by degrading the 3′ end to generate a fork.

Contrary to our original prediction of a presynaptic role of ExoI, our results suggest that ExoI can carry out a postsynaptic role on recombination intermediates where invasion has not occurred at the 3’ end, but elsewhere on the 3’ overhang and that RecJ carries out a presynaptic role affecting the location of this paranemic invasion (Fig 9). If this is so, we need to explain why, in bacteriophage lambda crosses, the presence of exonuclease I stimulates Chi promoted recombination when Chi lies opposite a heterology. Interestingly, it was shown that the frequency of Chi stimulated recombination diminished as a function of the length of heterology only when the Chi-containing parent of the lambda cross was in excess over the Chi free parent, arguing that the inhibition of recombination caused by the heterology was due to ‘distractor’ exchanges with the Chi containing parent [40]. Our results suggest that these ‘distractor’ exchanges occur with the 3’ single-strand after it has formed a potentially productive paranemic joint with the unbroken template for recombination. The absence of ExoI will prolong the life of this post-synaptic 3’ single-strand and increase the probability of ‘distractor’ exchanges, thereby reducing the frequency of productive recombination.

A potential reason for initiation of recombination via paranemic invasion by a region on the single-strand coated with RecA protein is provided in a recent model of homology searching [43]. This model proposes that a single-strand of a broken chromosome, coated with RecA protein is extended across the *E*. *coli* cell allowing it to interact with its unbroken homologue (which is not extended) via a two-dimensional, as opposed to a three dimensional, search. The model for homology searching implies that all regions along the RecA coated single-strand, and not just its 3’ end, are used to search for homology in the unbroken template. If this is correct, then invasions that are not at the 3’ end may result in intermediates such as those described in our model, and depicted in Fig 9(c), where the 3’ end protrudes from the D-loop and needs to be incorporated before repair synthesis can take place. We expect that there will be a distribution of invasion events, with some occurring at or close to the 3’ end and others occurring far away from the 3’ end. We propose that the events occurring further away from the 3’ end will be more frequent in the presence of RecJQ; all events, except for the small minority initiated precisely at the 3’ end, will be characterised by repair synthesis stimulated by Exo I.

## Materials and methods

### Bacterial strains, plasmids and oligonucleotides

All strains used are listed in S2 Table. The strains were derived from BW27784, which has been designed to ensure efficient and uniform induction of gene expression by arabinose and in which arabinose is not used as a carbon source [44]. The plasmids used in the construction of the strains and the oligonucleotides used in the construction of the plasmids are listed in S3 and S4 Tables.

### Isolation of chromosomal DNA in agarose plugs and psoralen crosslinking of the DNA

Unless otherwise indicated, overnight bacterial cultures were diluted to an OD_600nm_ of 0.02 in LB medium supplemented with 0.5% glucose. The cells were allowed to grow at 37°C to an OD_600nm_ of 0.2–0.3 and then re-diluted in LB with 0.5% glucose to an OD_600nm_ of 0.02. They were divided into two sub-cultures and left to grow for another 15 minutes. After that, 0.2% arabinose was added to one sub-culture in order to induce DSB formation mediated by SbcCD. An hour after the addition of arabinose, cells were harvested at 4°C and washed 3 times in TEN buffer (50 mM Tris, 50 mM EDTA, 100 mM NaCl, pH 8.0). After the final wash, cells were re-suspended in the calculated volume of TEN buffer to give a total cell OD_600nm_ of 40 and mixed with an equal volume of melted 0.8% (w/v) low melting point (LMP) agarose in distilled H_2_O. Immediately, the mixture was poured into plug moulds. Once set at 4°C, the plugs were incubated overnight at 37°C in proteinase K solution (1 mg/ml) dissolved in NDS buffer (0.5 M EDTA, 10 mM Tris, 0.55 M NaOH, 36.8 mM lauroyl sarcosine; pH 8.0; 1 ml/plug). On the next day, the solution was replaced with fresh proteinase K solution for another overnight incubation in NDS buffer. Finally, the plugs were stored at 4°C in fresh NDS buffer without proteinase K.

For crosslinking, after the cells were harvested at 4°C, the pellets were suspended in 1.6 ml of ice-cold PBS (137 mM NaCl, 2.7 mM KCl, 10 mM Na_2_HPO_4_ and 1.8 mM KH_2_PO_4_) and 0.4 ml of TMP stock solution (200 μg/ml TMP in 100% ethanol) was added to each cell suspension to a final concentration of 40 μg/ml. After addition of TMP, the cells were incubated for 10 minutes in the dark on ice. Afterwards, the cells were placed on precooled petri dishes, inside a UVP crosslinker on a precooled metal surface and irradiated at UV exposure setting of 200,000 μjoules/cm^2^. Following the irradiation, the cell suspensions were transferred back to Eppendorf tubes on ice and spun down. The pellets were then used for DNA extraction in agarose plugs as described above.

Before digestion of the DNA, a plug was washed in 1ml of TE buffer for 5 hours, replacing the buffer every hour. Before the 6th wash, each plug was divided into two halves with a clean scalpel. For the 6th wash, instead of TE buffer, 1 ml of 1X appropriate restriction buffer was used per plug and this wash took place at 37°C for 5 hours under gentle agitation. Once washed, the plugs were digested in 1 ml of fresh 1X appropriate restriction buffer with 100 U of enzyme per plug. Digestions were set overnight at 37°C under gentle rocking. Next day the digestion mixture was replaced with fresh buffer and fresh enzyme (100 U) and the reaction was carried out for another three hours.

### Native-native two-dimensional (2-D) agarose gel electrophoresis

2-D gels were performed as described in [15]. Briefly, an agarose plug containing digested DNA was run in the first dimension on a 0.4% (w/v) agarose gel in 1 x TBE (89 mM Tris-borate, 2 mM EDTA) at 2.3 V/cm for 30 hours at 4°C. The lane was cut out, rotated 90°, and set in the second dimension agarose (1% in 1 x TBE supplemented with 0.3 μg/ml of ethidium bromide). The second dimension was run at 3.6 V/cm for 14 hours at 4°C. The DNA was transferred to a positively charged nylon membrane (Roche Diagnostics GmbH, 11417240001) by Southern blotting and crosslinked using UV-light.

### Radioactive detection of DNA

DNA fragments were detected using ^32^P α-dATP incorporated radio-labelled DNA probes (prepared using Stratagene Prime-It II random primer labelling kit). Probes were hybridised to membranes overnight at 65°C in 10-15ml of hybridization buffer (7% SDS, 0.5 M NaH_2_PO_4_, 1 mM EDTA). Membranes were washed for 15 minutes at 60°C in 2X SSC (1X SSC: 0.15 M NaCl, 0.015 M Na-citrate) supplemented with 0.1% SDS and then 30 minutes in 0.5 x SSC supplemented with 0.1% SDS.

### Quantification of X-spike and Y-arc DNA structures

The percentage of the DNA in the form of HJs out of the total amount of DNA was determined by quantifying the intensities of the X-spikes and the n spots using ImageQuant software. For quantification, a grid was positioned over the spike and the median background signal emitted by the membrane was subtracted from the signal emitted by the DNA in each cell of the grid. The intensity of the n spot was calculated as the sum of the signal emitted from the whole spot. Following this, the percentage of DNA in the entire X-spike over the linear DNA was calculated by dividing the sum of the signals obtained from the grid by the sum of the grid and the n spot signal (Signal from X-spike/ (Signal from X-spike + signal from n)). Estimated contamination from the Y-arc was also removed from the calculations as accurately as possible.

For the quantification of Y-arc, a polygon area was drawn encompassing the entire Y-arc. And the background signal was subtracted from the signal emitted from the Y-arc area. Afterwards, the signal obtained from the Y-arc was divided by the sum of the Y-arc and the n spot signal and thus the percentage of DNA in the Y-arc out of the total amount of DNA was calculated.

### RecA ChIP-Seq

RecA ChIP-Seq was carried out as described [16]. Briefly, cells were grown as described above. RecA-DNA interactions were chemically crosslinked with formaldehyde (Sigma-Aldrich, at a final concentration of 1%) for 10 minutes at 22.5°C. Crosslinking was quenched by the addition of 0.5 M glycine (Sigma-Aldrich). Cells were collected by centrifugation at 1,500 x g for 10 minutes and then washed three times in ice-cold 1X PBS. The pellet was then re-suspended in 250 μl ChIP buffer (200 mM Tris-HCl (pH 8.0), 600 mM NaCl 4% Triton X, Complete protease inhibitor cocktail EDTA-free (Roche)). Sonication of crosslinked samples was performed using the Diagenode Bioruptor at 30 seconds intervals for 10 minutes at high amplitude. After sonication, 350 μl of ChIP buffer was added to each sample, the samples were mixed by gentle pipetting and 100 μl of each lysate were removed and stored as ‘input’. Immunoprecipitation was performed overnight at 4°C using 1/100 anti-RecA antibody (Abcam, ab63797). Immunoprecipitated (IP) samples were then incubated with Protein G Dynabeads (Life Technologies) for 2 hours with rotation at room temperature. All samples were washed three times with 1 X PBS + 0.02% Tween-20 before re-suspending the Protein G Dynabeads in 200 μl of TE buffer + 1% SDS. 100 μl of TE buffer were added to the input samples and all samples were then incubated at 65°C for 10 hours to reverse the formaldehyde cross-links. DNA was isolated using the MinElute PCR purification kit (Qiagen) according to manufacturer’s instructions. DNA was eluted in 100 μl of TE buffer using a 2-step elution. Samples were stored at -20°C. ChIP libraries were prepared for high-throughput sequencing following NEB’s protocol from the NEBNext ChIP-Seq library preparation kit. Briefly, ChIP-enriched DNAs were subjected to end repair to fill in ssDNA overhangs, remove 3’ phosphates and phosphorylate the 5’ ends of sheared DNA. Klenow exo- was used to adenylate the 3’ ends of the DNA and NEBNext adaptor were ligated using T4 DNA ligase. After each step, the DNA was purified using the Qiagen MinElute PCR purification kit according to the manufacturer’s instructions. After adaptor ligation, the adaptor-modified DNA fragments were enriched by PCR using primers corresponding to the beginning of each adaptor. Finally, agarose gel electrophoresis was used to size select adaptor-ligated DNA with an average size of approximately 275 bp. All samples were quantified on a Bioanalyzer (Agilent) before being sequenced on the Illumina HiSeq 4000 by Edinburgh Genomics. The analysis was performed as described in [45]. Briefly, 75bp pair-end reads were mapped to the DL5216 draft reference genome sequence using the default parameters of software Bowtie 2 [46]. The distribution of reads along the *E*. *coli* genome was visualized using the Integrated Genome Browser [47].

## Supporting information

S1 Fig 2-D gels of the crosslinked DNA fragments from Δ*ruvAB* strains. A) 2-D gels of the crosslinked 18-24kb OP, 24-30kb OP, Chi-9kb OD, 9-15kb OD, 18-24kb OD and 24-30kb OD fragments from Δ*ruvAB* strains containing the palindrome, grown in the presence of 0.2% arabinose for 60 minutes. B) 2-D gels of the crosslinked central, Chi-9kb OP, 9-15kb OP,18-24kb OP, 24-30kb OP, Chi-9kb OD, 9-15kb OD, 18-24kb OD and 24-30kb OD fragments from Δ*ruvAB* strains containing the palindrome, grown in the presence of glucose. The strains used were DL7272 (Δ*ruvAB* Chi-Chi), DL7253 (Δ*ruvAB* Chi-9kb OP), DL7259 (Δ*ruvAB* 9-15kb OP), DL7270 (Δ*ruvAB* 18-24kb OP), DL7271 (Δ*ruvAB* 24-30kb OP), DL7251 (Δ*ruvAB* Chi-9kb OD), DL7258 (Δ*ruvAB* 9-15kb OD), DL7261 (Δ*ruvAB* 18-24kb OD) and DL7262 (Δ*ruvAB* 24-30kb OD). OP and OD mean origin-proximal and origin-distal sides.
(PPTX)

S2 Fig 2-D gels of the non-crosslinked DNA fragments from Δ*ruvAB* strains. 2-D gels of the non-crosslinked 18-24kb OP, 24-30kb OP, Chi-9kb OD, 9-15kb OD, 18-24kb OD and 24-30kb OD fragments from Δ*ruvAB* strains containing the palindrome, grown in the presence of 0.2% arabinose for 60 minutes. The strains used were DL7270 (Δ*ruvAB* 18-24kb OP), DL7271 (Δ*ruvAB* 24-30kb OP), DL7251 (Δ*ruvAB* Chi-9kb OD), DL7258 (Δ*ruvAB* 9-15kb OD), DL7261 (Δ*ruvAB* 18-24kb OD) and DL7262 (Δ*ruvAB* 24-30kb OD). OP and OD mean origin-proximal and origin-distal sides.
(PPTX)

S3 Fig Accumulation of Y-arc across the 9-15kb non-crosslinked DNA fragment on the origin-proximal side of the DSB under different conditions. The DNA containing agarose plugs were treated under different conditions to test the stability of the Y-arc that accumulates in the non-crosslinked DNA fragment. The conditions included the normal one (incubation in the restriction enzyme buffer containing high salt), incubation in TE buffer at 37°C for 3 hours or for overnight and incubation in TE buffer at 45°C for 3 hours.
(PPTX)

S4 Fig Percentage of X-spike across the central fragment and the Chi-9kb and 9-15kb fragments on the origin-proximal side of the DSB isolated from Δ*ruvAB* single mutant and Δ*xonA* Δ*ruvAB* double mutant strains grown in presence of arabinose. Quantification of the intensities of the repair forks generated in the crosslinked DNA samples represented as percentage of DNA in the X-spike out of the total 6kb DNA fragments. OP and OD mean origin-proximal and origin-distal sides. Error bars represent the standard error of the mean where n = 3. The strains used were DL7840 (Δ*xonA* Δ*ruvAB* 9-15kb OP), DL7839 (Δ*xonA* Δ*ruvAB* Chi-9kb OP), DL7841 (Δ*xonA* Δ*ruvAB* Chi-Chi), DL7259 (Δ*ruvAB*, 9-15kb OP), DL7253 (Δ*ruvAB*, Chi-9kb OP) and DL7272 (Δ*ruvAB*, Chi-Chi).
(PPTX)

S5 Fig Percentage of X-spike across the central fragment and the Chi-9kb and 9-15kb fragments on the origin-proximal side of the DSB isolated from Δ*ruvAB* single mutant, Δ*recJ* Δ*ruvAB and* Δ*recQ* Δ*ruvAB* double mutant strains grown in presence of arabinose. Quantification of the intensities of the repair forks generated in the crosslinked DNA samples represented as percentage of DNA in the X-spike out of the total 6kb DNA fragments. OP and OD mean origin-proximal and origin-distal sides. Error bars represent the standard error of the mean where n = 3. The strains used were DL7859 (Δ*recJ* Δ*ruvAB*, 9-15kb OP), DL7857 (Δ*recJ* Δ*ruvAB*, Chi-9kb OP), DL7827 (Δ*recJ* Δ*ruvAB*, Chi-Chi), DL7591 (Δ*recQ* Δ*ruvAB*, 9-15kb OP), DL7874 (Δ*recQ* Δ*ruvAB*, Chi-9kb OP), DL7588 (Δ*recQ* Δ*ruvAB*, Chi-Chi), DL7259 (Δ*ruvAB*, 9-15kb OP), DL7253 (Δ*ruvAB*, Chi-9kb OP) and DL7272 (Δ*ruvAB*, Chi-Chi).
(PPTX)

S1 Table Percentage of X-spike, Y-arc and the ratio of X-spike to Y-arc for cultures grown in M9 minimal medium for 0.8 generation time and 1.6 generation times. (DOCX)

S2 Table Bacterial strains used in this study. (DOCX)

S3 Table Plasmids used in this study. (DOCX)

S4 Table DNA oligonucleotide sequences used in this study. (DOCX)
