# Supplementary figures and images for: Distribution of Holliday junctions and repair forks during Escherichia coli DNA double-strand break repair
Source: PLoS Genet. 2021 Aug 25;17(8):e1009717. doi: 10.1371/journal.pgen.1009717 (PMC8386832; doi:10.1371/journal.pgen.1009717)

## Slide 1
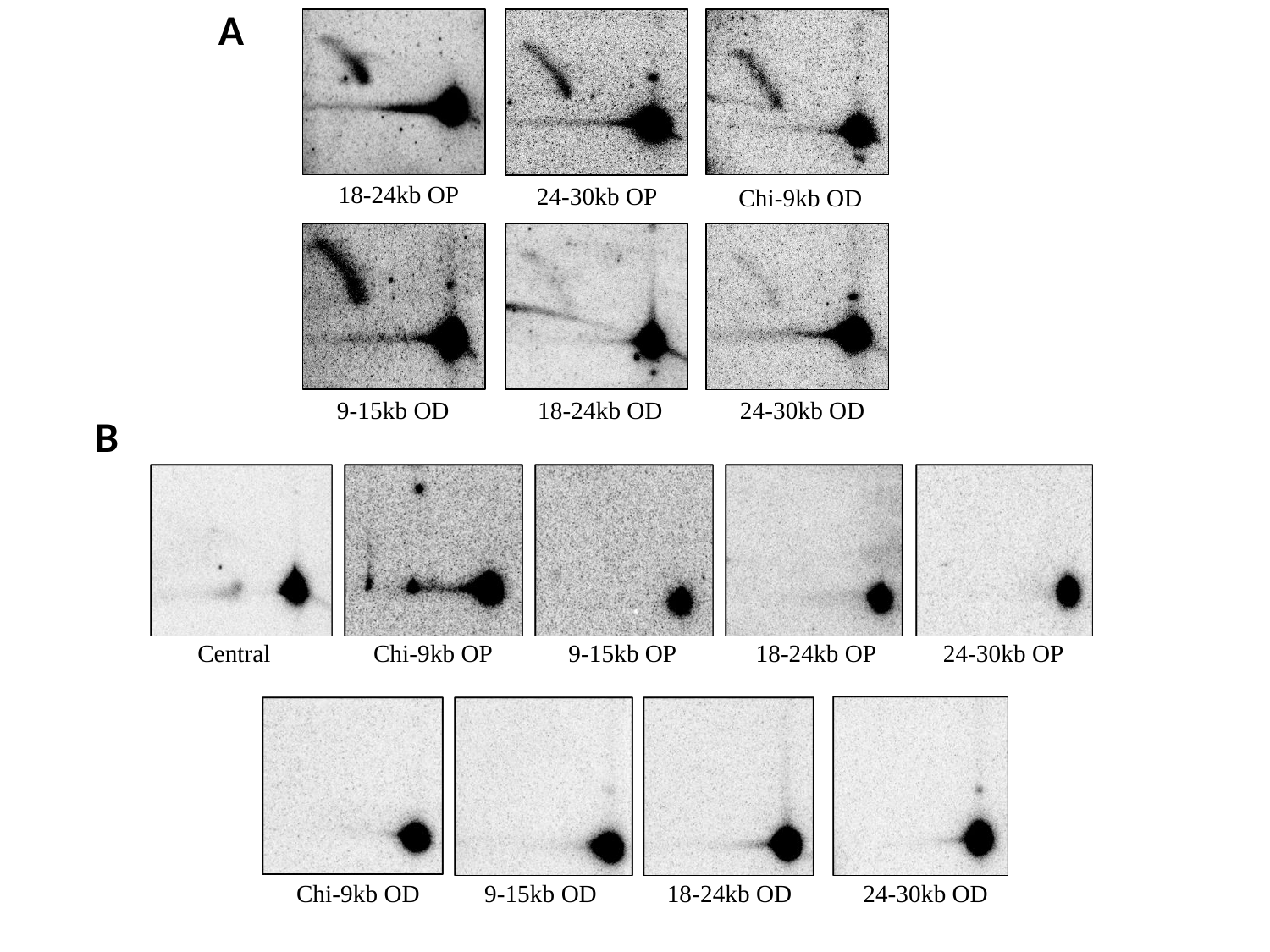

A
18-24kb OP
24-30kb OP
Chi-9kb OD
9-15kb OD
18-24kb OD
24-30kb OD
B
Central
Chi-9kb OP
 9-15kb OP
18-24kb OP
24-30kb OP
Chi-9kb OD
9-15kb OD
18-24kb OD
24-30kb OD

Supplement: S1 Fig — A) 2-D gels of the crosslinked 18-24kb OP, 24-30kb OP, Chi-9kb OD, 9-15kb OD, 18-24kb OD and 24-30kb OD fragments from ΔruvAB strains containing the palindrome, grown in the presence of 0.2% arabinose for 60 minutes. B) 2-D gels of the crosslinked central, Chi-9kb OP, 9-15kb OP,18-24kb OP, 24-30kb OP, Chi-9kb OD, 9-15kb OD, 18-24kb OD and 24-30kb OD fragments from ΔruvAB strains containing the palindrome, grown in the presence of glucose. The strains used were DL7272 (ΔruvAB Chi-Chi), DL7253 (ΔruvAB Chi-9kb OP), DL7259 (ΔruvAB 9-15kb OP), DL7270 (ΔruvAB 18-24kb OP), DL7271 (ΔruvAB 24-30kb OP), DL7251 (ΔruvAB Chi-9kb OD), DL7258 (ΔruvAB 9-15kb OD), DL7261 (ΔruvAB 18-24kb OD) and DL7262 (ΔruvAB 24-30kb OD). OP and OD mean origin-proximal and origin-distal sides. (PPTX) [file pgen.1009717.s001.pptx]

## Slide 1
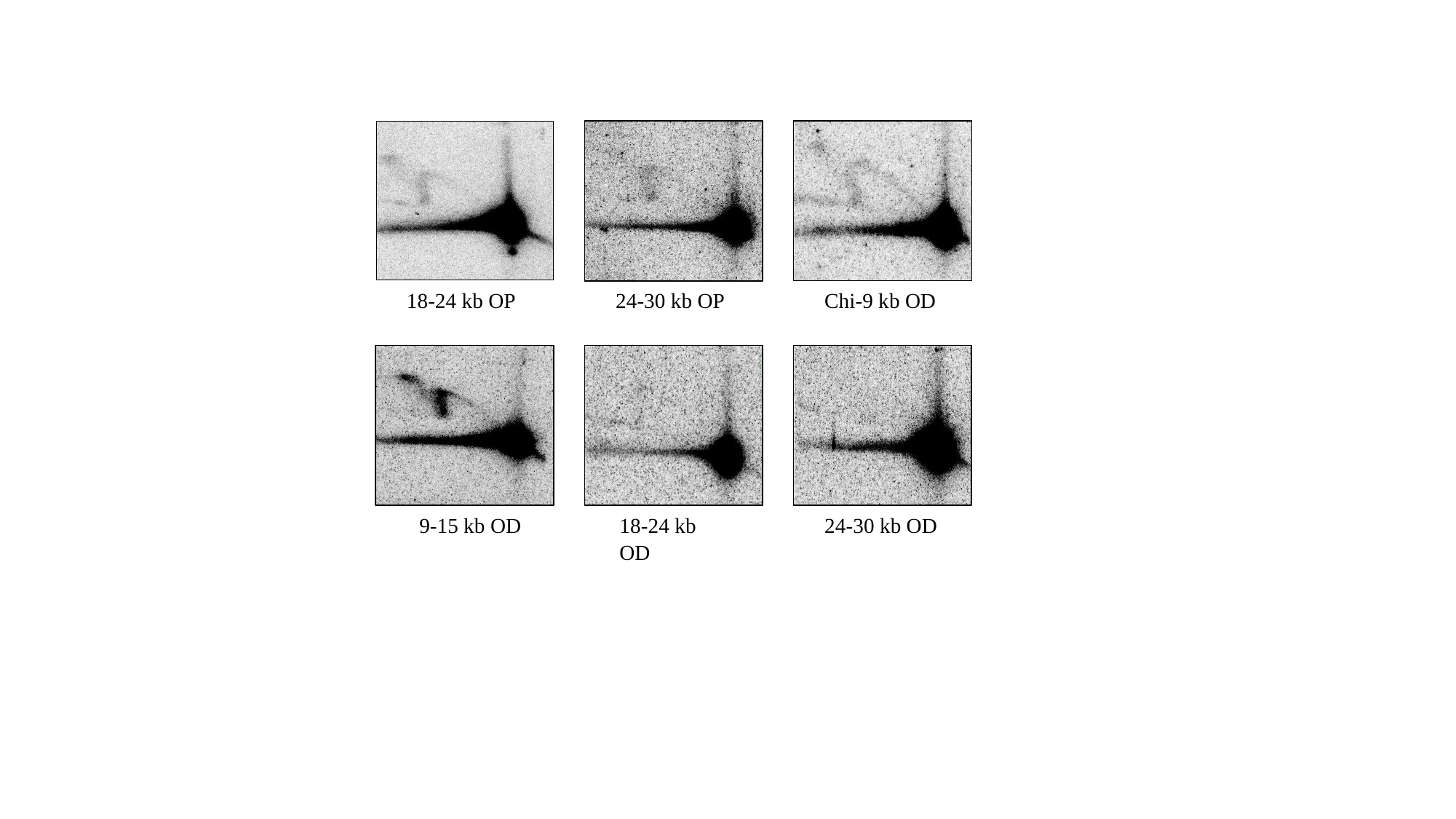

18-24 kb OP
24-30 kb OP
Chi-9 kb OD
9-15 kb OD
18-24 kb OD
24-30 kb OD

Supplement: S2 Fig — 2-D gels of the non-crosslinked 18-24kb OP, 24-30kb OP, Chi-9kb OD, 9-15kb OD, 18-24kb OD and 24-30kb OD fragments from ΔruvAB strains containing the palindrome, grown in the presence of 0.2% arabinose for 60 minutes. The strains used were DL7270 (ΔruvAB 18-24kb OP), DL7271 (ΔruvAB 24-30kb OP), DL7251 (ΔruvAB Chi-9kb OD), DL7258 (ΔruvAB 9-15kb OD), DL7261 (ΔruvAB 18-24kb OD) and DL7262 (ΔruvAB 24-30kb OD). OP and OD mean origin-proximal and origin-distal sides. (PPTX) [file pgen.1009717.s002.pptx]

## Slide 1
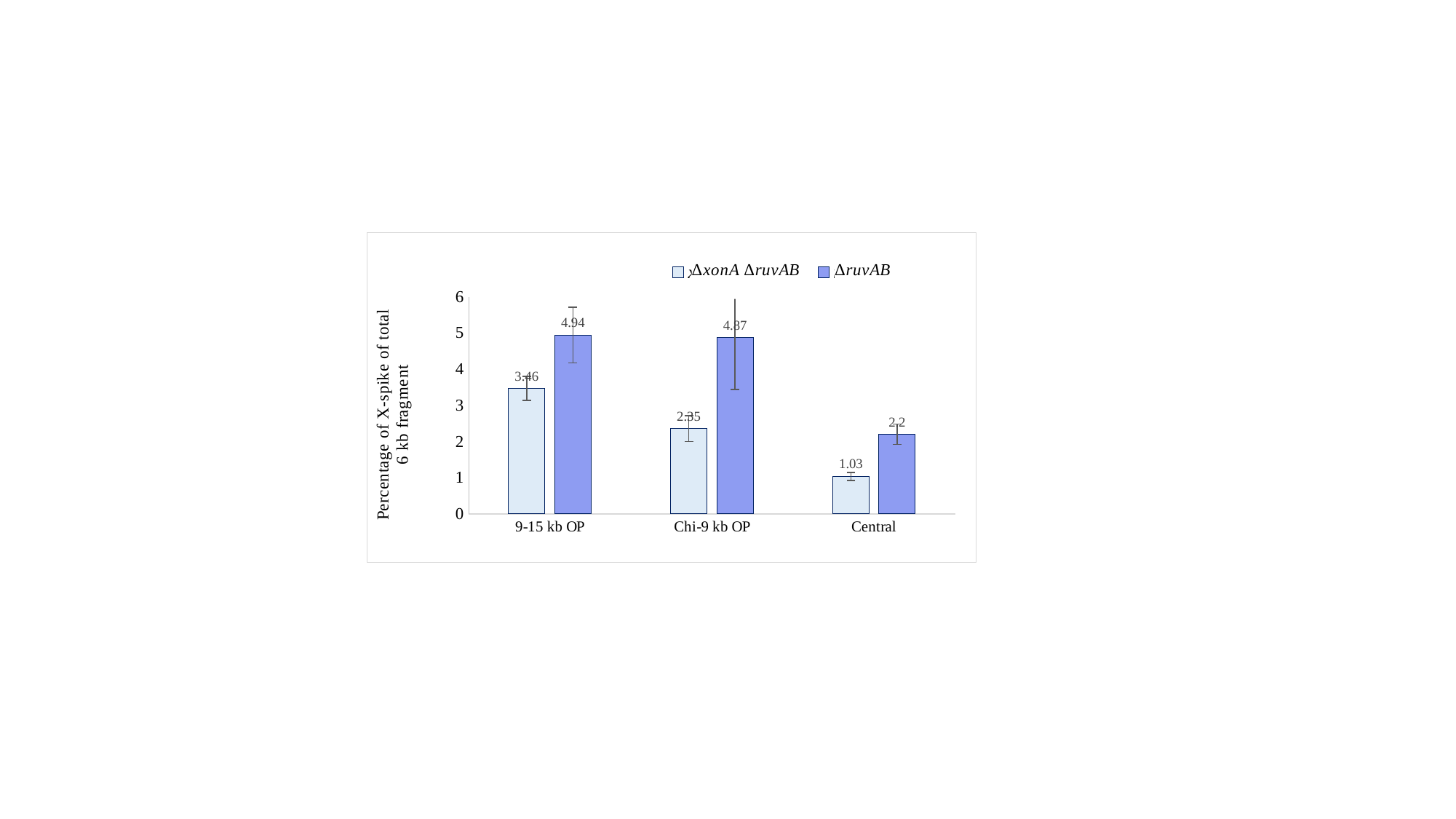

### Chart
| Category | xonA ruvAB | ruvAB |
|---|---|---|
| 9-15 kb OP | 3.4615444622043414 | 4.94 |
| Chi-9 kb OP | 2.353136626408776 | 4.87 |
| Central | 1.031399606299378 | 2.2 |

Supplement: S4 Fig — Quantification of the intensities of the repair forks generated in the crosslinked DNA samples represented as percentage of DNA in the X-spike out of the total 6kb DNA fragments. OP and OD mean origin-proximal and origin-distal sides. Error bars represent the standard error of the mean where n = 3. The strains used were DL7840 (ΔxonA ΔruvAB 9-15kb OP), DL7839 (ΔxonA ΔruvAB Chi-9kb OP), DL7841 (ΔxonA ΔruvAB Chi-Chi), DL7259 (ΔruvAB, 9-15kb OP), DL7253 (ΔruvAB, Chi-9kb OP) and DL7272 (ΔruvAB, Chi-Chi). (PPTX) [file pgen.1009717.s004.pptx]

## Slide 1
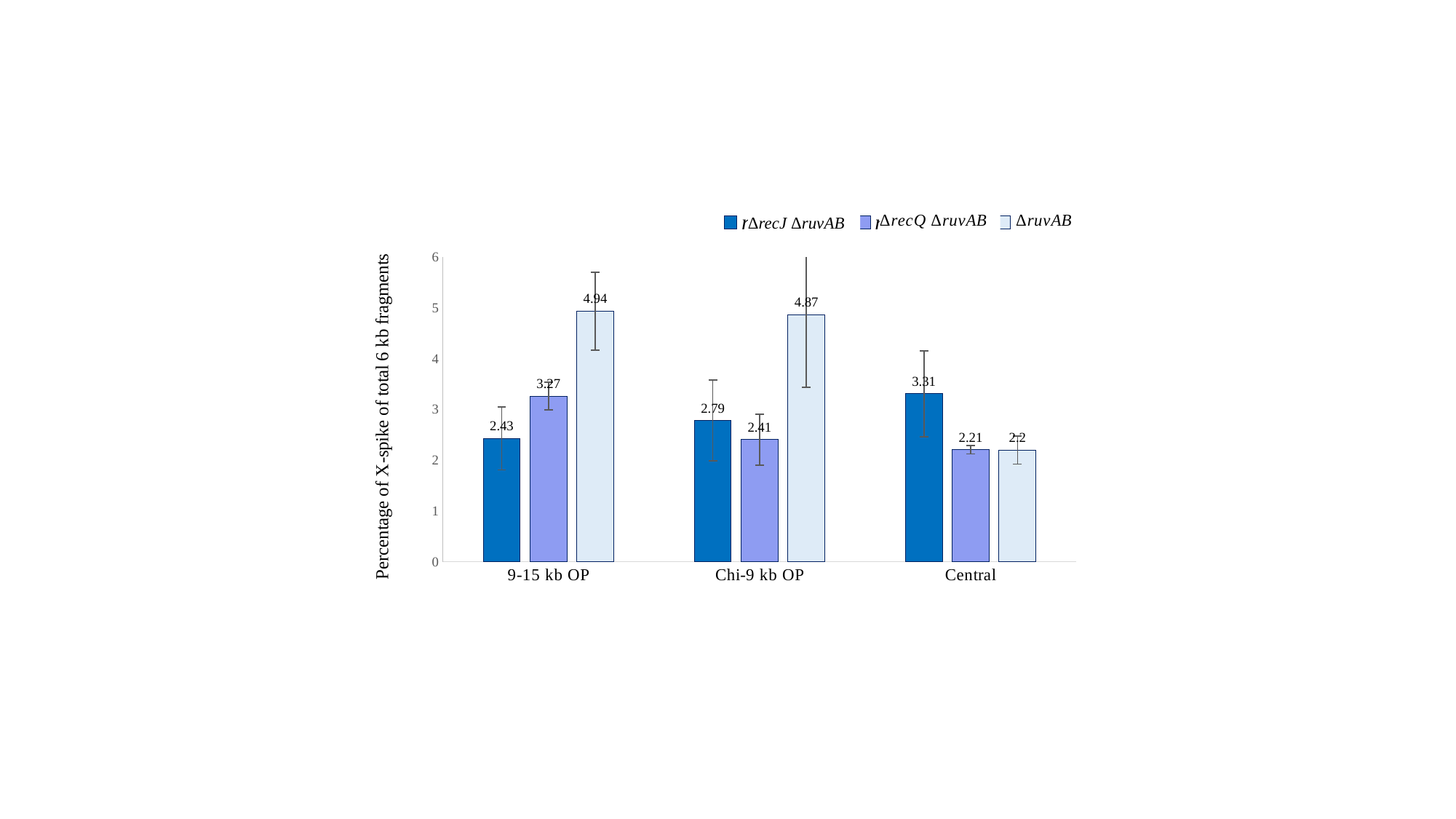

### Chart
| Category | recJ ruvAB | | ruvAB |
|---|---|---|---|
| 9-15 kb OP | 2.431169117122285 | 3.266666666666666 | 4.94 |
| Chi-9 kb OP | 2.785402790811313 | 2.4060053040673712 | 4.87 |
| Central | 3.3106856881743383 | 2.2050188318931903 | 2.2 |ΔrecJ ΔruvAB

Supplement: S5 Fig — Quantification of the intensities of the repair forks generated in the crosslinked DNA samples represented as percentage of DNA in the X-spike out of the total 6kb DNA fragments. OP and OD mean origin-proximal and origin-distal sides. Error bars represent the standard error of the mean where n = 3. The strains used were DL7859 (ΔrecJ ΔruvAB, 9-15kb OP), DL7857 (ΔrecJ ΔruvAB, Chi-9kb OP), DL7827 (ΔrecJ ΔruvAB, Chi-Chi), DL7591 (ΔrecQ ΔruvAB, 9-15kb OP), DL7874 (ΔrecQ ΔruvAB, Chi-9kb OP), DL7588 (ΔrecQ ΔruvAB, Chi-Chi), DL7259 (ΔruvAB, 9-15kb OP), DL7253 (ΔruvAB, Chi-9kb OP) and DL7272 (ΔruvAB, Chi-Chi). (PPTX) [file pgen.1009717.s005.pptx]
