# Supplementary material for: Distribution of Holliday junctions and repair forks during Escherichia coli DNA double-strand break repair
Source: PLoS Genet. 2021 Aug 25;17(8):e1009717. doi: 10.1371/journal.pgen.1009717 (PMC8386832; doi:10.1371/journal.pgen.1009717)
Supplement: S3 Fig — The DNA containing agarose plugs were treated under different conditions to test the stability of the Y-arc that accumulates in the non-crosslinked DNA fragment. The conditions included the normal one (incubation in the restriction enzyme buffer containing high salt), incubation in TE buffer at 37°C for 3 hours or for overnight and incubation in TE buffer at 45°C for 3 hours. (PPTX) [file pgen.1009717.s003.pptx]

## Slide 1
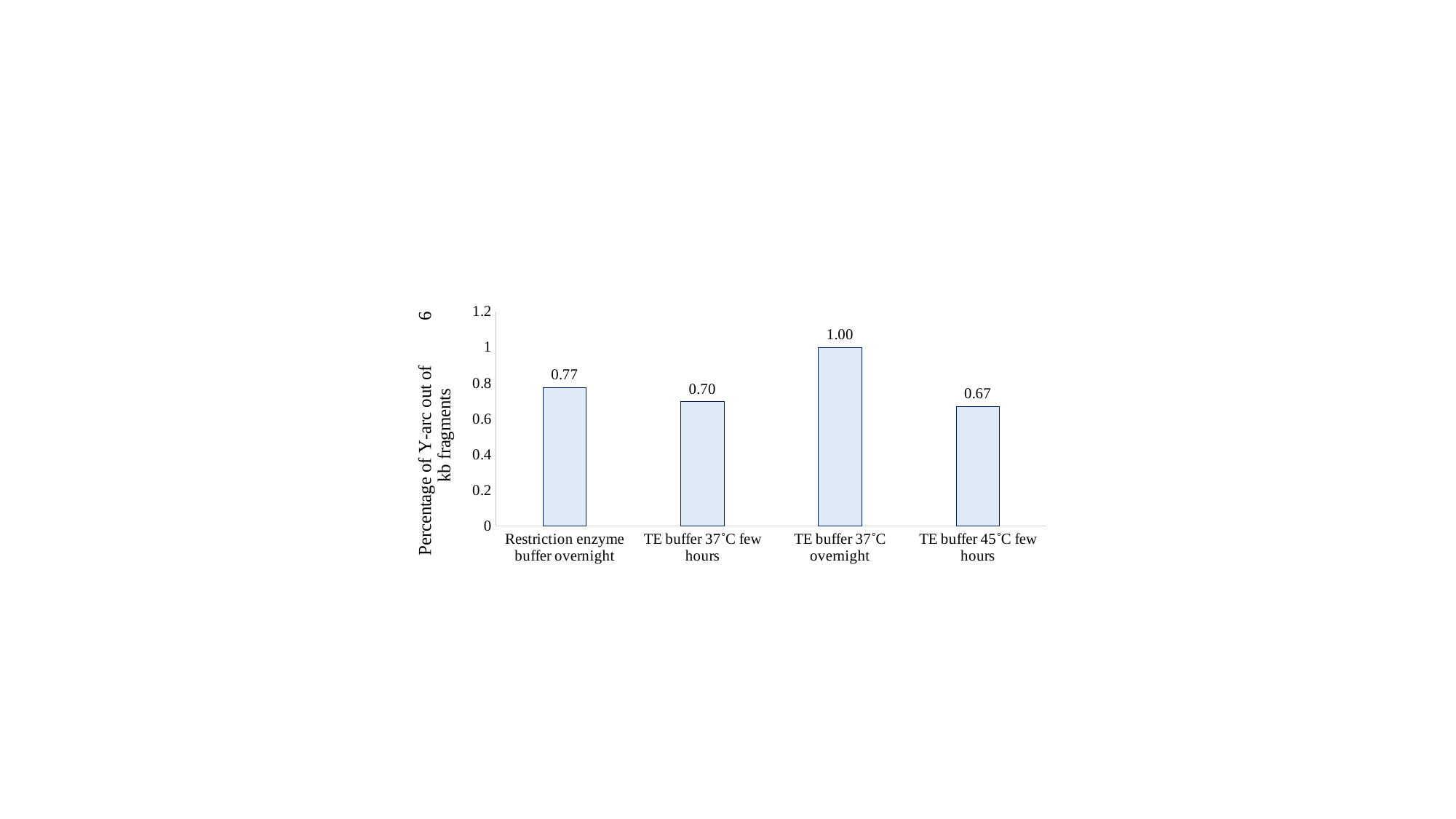

### Chart
| Category | |
|---|---|
| Restriction enzyme buffer overnight | 0.774388732126197 |
| TE buffer 37˚C few hours | 0.695928958753429 |
| TE buffer 37˚C overnight | 0.997737042741977 |
| TE buffer 45˚C few hours | 0.6683610033840026 |
