# Supplementary material for: Distribution of Holliday junctions and repair forks during Escherichia coli DNA double-strand break repair
Source: PLoS Genet. 2021 Aug 25;17(8):e1009717. doi: 10.1371/journal.pgen.1009717 (PMC8386832; doi:10.1371/journal.pgen.1009717)
Supplement: S1 Table — (DOCX) [file pgen.1009717.s006.docx]

| Cell culture condition | Average percentage of X-spike | Standard Error of Mean (SEM) of X-spike percentage | Average percentage of Y-arc | Standard Error of Mean (SEM) of Y-arc percentage | Ratio of X-spike to Y-arc |
| --- | --- | --- | --- | --- | --- |
| M9 minimal medium with 0.2% arabinose for less than a single generation time (0.8 generation time) | 1.43 | 0.12 | 0.35 | 0.01 | 4.08 |
|  |  |  |  |  |  |
|  |  |  |  |  |  |
| M9 minimal medium with 0.2% arabinose for 1.6 generation times | 2.05 | 0.10 | 0.35 | 0.02 | 5.85 |
|  |  |  |  |  |  |
|  |  |  |  |  |  |

**S1 Table: Percentage of X-spike, Y-arc and the ratio of X-spike to Y-arc for cultures grown in M9 minimal medium for 0.8 generation time and 1.6 generation times.**
