# Supplementary material for: Distribution of Holliday junctions and repair forks during Escherichia coli DNA double-strand break repair
Source: PLoS Genet. 2021 Aug 25;17(8):e1009717. doi: 10.1371/journal.pgen.1009717 (PMC8386832; doi:10.1371/journal.pgen.1009717)
Supplement: S2 Table — (DOCX) [file pgen.1009717.s007.docx]

**S2 Table:** **Bacterial strains used in this study.**

| Strain | Genotype | Source |
| --- | --- | --- |
| BW27784 | Δ*(araD-araB)567* Δ*(araH-araF)570(*::*FRT)* Δ*araEp-532*::*FRT φPcp18araE533* Δ*(rhaD-rhaB)568 hsdR514*Δ*lacZ478(*::*rrnB-3)* | [1] |
| DL4311 | BW27784 Δ*recG263*::*Km mhpA*::3xChi *lacZY*:: 3xChi *proA*::I-SceI_cs_ *tsx*::I-SceI_cs_ *P_araBAD_-sbcDC lacZ*::Pal246 *cynX*::Gm^R^ *lacIq lacZ*ΔChi | [2] |
| DL5216 | *mhpA*::3xChi *lacZY*::3xChi *tsx*::I-SceI_cs_ *proA*::I-SceI_cs_ *P_araBAD_-sbcDC lacZ*::Pal246 *cynX*::Gm^R^ *lacIq lacZχ- rph+ cynX* ΔChi *codB*ΔChi *prpE*ΔChi *mhpC*ΔChi | This work |
| DL 5670 | BW27784 *mhpA*::3xChi *lacZY*::3xChi *P_araBAD_-sbcDC lacZ*::Pal246 *cynX*::Gm^R^ *lacIq lacZ*ΔChi *rph^+^ cynX*ΔChi *codB*ΔChi *prpE*ΔChi *mhpC*ΔChi | This work |
| DL7203 | DL5670 ΔGm^R^ cassette promoter | PMGR using pDL6965 |
| DL7251 | DL7203 Δ*ruvAB mhpA*::NotI_cs_ *mhpE.mhpT*::NotI_cs_ | PMGR using pDL2757, pDL6962 and pDL6964 |
| DL7253 | DL7203 Δ*ruvAB codA.cynR*::NotI_cs_ *lacZY*::NotI_cs_ | PMGR using pDL2757, pDL6966 and pDL7015 |
| DL7258 | DL7203 Δ*ruvAB yaiX*::NotI_cs_ *mhpE.mhpT*::NotI_cs_ | PMGR using pDL2757, pDL7209 and pDL6964 |
| DL7259 | DL7203 Δ*ruvAB prpC*::NotI_cs_ *codA.cynR*::NotI_cs_ | PMGR using pDL2757, pDL7177 and pDL6966 |
| DL7261 | DL7203 Δ*ruvAB yaiT*::NotI_cs_ *yaiS*::NotI_cs_ | PMGR using pDL2757, pDL7214 and pDL7213 |
| DL7262 | DL7203 Δ*ruvAB ampH.sbmA*::NotI_cs_ *yaiT*::NotI_cs_ | PMGR using pDL2757, pDL7215 and pDL7214 |
| DL7270 | DL7203 Δ*ruvAB prpR*::NotI_cs_ *yahI.yahJ*::NotI_cs_ | PMGR using pDL2757, pDL7245 and pDL7246 |
| DL7271 | DL7203 Δ*ruvAB yahC.yahD*::NotI_cs_ *yahI.yahJ*::NotI_cs_ | PMGR using pDL2757, pDL7247 and pDL7246 |
| DL7272 | DL7203 Δ*ruvAB mhpA*::NotI_cs_ *lacZY*::NotI_cs_ | PMGR using pDL2757, pDL6962 and pDL7015 |
| DL7577 | DL7203 *ydeJ*::NotI_cs_ Δ*ruvAB prpC*::NotI_cs_ *codA.cynR*::NotI_cs_ | PMGR using pDL7565, pDL2757, pDL7177 and pDL6966 |
| DL7588 | DL7203 Δ*ruvAB* Δ*recQ mhpA::*NotI_cs_ *lacZY*::NotI_cs_ | PMGR using pDL2757, pDL2765, pDL6962 and pDL7015 |
| DL7591 | DL7203 Δ*ruvAB* Δ*recQ prpC*::*NotI_cs_ codA.cynR*::NotI_cs_ | PMGR using pDL2757, pDL2765, pDL7177 and pDL6966 |
| DL7667 | DL7203 Δ*recQ* Δ*radA* *priA300* Δ*recG263*::Km^R^ Δ*ruvAB mhpA*::NotI_cs_ *lacZY*::NotI_cs_ | PMGR using pDL2765, pDL4428, pDL4947, pDL2757, pDL6962, pDL7015 and P1 using DL4311 |
| DL7827 | DL7203 Δ*recJ* Δ*ruvAB mhpA*::*NotI_cs_ lacZY*::NotI_cs_ | PMGR using pDL2757, pDL2713, pDL6962 and pDL7015 |
| DL7839 | DL7203 Δ*xonA* Δ*ruvAB codA.cynR*::*NotI_cs_ lacZY*::NotI_cs_ | PMGR using pDL2757, pDL2745, pDL6966 and pDL7015 |
| DL7840 | DL7203 Δ*xonA* Δ*ruvAB prpC*::*NotI_cs_ codA.cynR*::NotI_cs_ | PMGR using pDL2757, pDL2745, pDL7177 and pDL6966 |
| DL7841 | DL7203 Δ*xonA* Δ*ruvAB mhpA*::*NotI_cs_ lacZY*::NotI_cs_ | PMGR using pDL2757, pDL2745, pDL6962 and pDL7015 |
| DL7857 | DL7203 Δ*recJ* Δ*ruvAB codA.cynR*::*NotI_cs_ lacZY*::NotI_cs_ | PMGR using pDL2757, pDL2713, pDL6966 and pDL7015 |
| DL7859 | DL7203 Δ*recJ* Δ*ruvAB prpC*::*NotI_cs_ codA.cynR*::NotI_cs_ | PMGR using pDL2757, pDL2713, pDL7177 and pDL6966 |
| DL7874 | DL7203 Δ*ruvAB* Δ*recQ codA.cynR*::*NotI_cs_ lacZY*::NotI_cs_ | PMGR using pDL2757, pDL2765, pDL6966 and pDL7015 |

**References**

1. Khlebnikov A, Datsenko KA, Skaug T, Wanner BL, Keasling JD. Homogeneous expression of the P(BAD) promoter in Escherichia coli by constitutive expression of the low-affinity high-capacity AraE transporter. Microbiology (Reading). 2001;147(Pt 12):3241-7.

2. Mawer JS, Leach DR. Branch migration prevents DNA loss during double-strand break repair. PLoS Genet. 2014;10(8):e1004485.
