# Supplementary material for: Distribution of Holliday junctions and repair forks during Escherichia coli DNA double-strand break repair
Source: PLoS Genet. 2021 Aug 25;17(8):e1009717. doi: 10.1371/journal.pgen.1009717 (PMC8386832; doi:10.1371/journal.pgen.1009717)
Supplement: S3 Table — (DOCX) [file pgen.1009717.s008.docx]

**S3 Table:** **Plasmids used in this study.**

| **Plasmid** | **Description** | **Source** |
| --- | --- | --- |
| pDL1605 | pTOF24 plasmid; Cm^R^ Km^R^ Ts Suc^S^ | [1] |
| pDL2713 | pTOF24 Δ*recJ*; Cm^R^ Ts Suc^S^ | Ewa Okely, Leach Lab |
| pDL2745 | pTOF24 Δ*xonA*; Cm^R^ Ts Suc^S^ | [2] |
| pDL2757 | pTOF24 Δ*ruvAB*; Cm^R^ Ts Suc^S^ | [3] |
| pDL2765 | pTOF24 Δ*recQ*; Cm^R^ Ts Suc^S^ | This work |
| pDL4428 | pTOF24 Δ*radA*; Cm^R^ Ts Suc^S^ | This work |
| pDL4947 | pTOF24 *priA300*; Cm^R^ Ts Suc^S^ | [4] |
| pDL6962 | pTOF24 *mhpA*::NotI_cs_ (3kb OD from palindrome between palindrome and Chi array); Cm^R^ Ts Suc^S^ | This work |
| pDL6964 | pTOF24 *mhpE.mhpT*::NotI_cs_ (9kb OD from palindrome); Cm^R^ Ts Suc^S^ | This work |
| pDL6965 | pTOF24 *cynX*::ΔGm^R^ promoter; Cm^R^ Ts Suc^S^ | This work |
| pDL6966 | pTOF24 *codA.cynR*::NotI_cs_ (9kb OP from palindrome); Cm^R^ Ts Suc^S^ | This work |
| pDL7015 | pTOF24 *lacZY*::NotI_cs_ (3kb OP from palindrome between Chi array and palindrome); Cm^R^ Ts Suc^S^ | This work |
| pDL7177 | pTOF24 *prpC*::NotI_cs_ (15kb OP from palindrome); Cm^R^ Ts Suc^S^ | This work |
| pDL7209 | pTOF24 *yaiX*::NotI_cs_ (15kb OD from palindrome); Cm^R^ Ts Suc^S^ | This work |
| pDL7213 | pTOF24 *yaiS*::NotI_cs_ (18kb OD from palindrome); Cm^R^ Ts Suc^S^ | This work |
| pDL7214 | pTOF24 *yaiT*::NotI_cs_ (24 kb OD from palindrome); Cm^R^ Ts Suc^S^ | This work |
| pDL7215 | pTOF24 *ampH.sbmA*::NotI_cs_ (30kb OD from palindrome); Cm^R^ Ts Suc^S^ | This work |
| pDL7245 | pTOF24 *prpR*::NotI_cs_ (18kb OP from palindrome); Cm^R^ Ts Suc^S^ | This work |
| pDL7246 | pTOF24 *yahI.yahJ*::NotI_cs_ (24kb OP from palindrome); Cm^R^ Ts Suc^S^ | This work |
| pDL7247 | pTOF24 *yahC.yahD*::NotI_cs_ (30kb OP from palindrome); Cm^R^ Ts Suc^S^ | This work |
| pDL7565 | pTOF *ydeJ*::NotI_cs_ (inserted to generate 12kb fragment); Cm^R^ Ts Suc^S^ | This work |

Cm^R^ - resistant to chloramphenicol; Km^R^ - resistant to kanamycin; Ts – temperature-sensitive; Suc^S^ - sensitive to products of sucrose degradation, Amp^R^ – resistant to ampicillin.

**References**

1. Merlin C, McAteer S, Masters M. Tools for characterization of *Escherichia coli* genes of unknown function. J Bacteriol. 2002;184(16):4573-4581.

2. Darmon E, Eykelenboom JK, Lincker F, Jones LH, White M, Okely E, Blackwood JK, Leach DRF. *E. coli* SbcCD and RecA control chromosomal rearrangement induced by an interrupted palindrome. Molecular Cell. 2010; 39(1):59-70.

3. White MA, Eykelenboom JK, Lopez-Vernaza MA, Wilson E, Leach DRF. Non-random segregation of sister chromosomes in *Escherichia coli*. Nature. 2008;455(7217):1248-1250.

4. Azeroglu B, Mawer JS, Cockram CA, White MA, Hasan AM, Filatenkova M, Leach DRF. RecG Directs DNA Synthesis during Double-Strand Break Repair. PLoS Genet. 2016;12(2):e1005799.
