# Supplementary material for: Distribution of Holliday junctions and repair forks during Escherichia coli DNA double-strand break repair
Source: PLoS Genet. 2021 Aug 25;17(8):e1009717. doi: 10.1371/journal.pgen.1009717 (PMC8386832; doi:10.1371/journal.pgen.1009717)
Supplement: S4 Table — (DOCX) [file pgen.1009717.s009.docx]

**S4 Table:** **DNA oligonucleotide sequences used in this study.**

| Name | Sequence | Purpose |
| --- | --- | --- |
| ΔGm^R^cassetteF1 | AAAAACTGCAGTCCTTAGGTGGCGGTACTTG | Crossover PCR to make ΔGm^R^ cassette promoter fragment for the construction of pDL6965 for the deletion of the promoter region of the Gm^R^ cassette |
| ΔGm^R^cassetteR1 | CCAGGCGAGTGTTTGATATTGATCTTTTCGGTCGTGAG |  |
| ΔGm^R^cassetteF2 | GATCAATATCAAACACTCGCCTGGTGGGCGCTGCCTGC |  |
| ΔGm^R^cassetteR2 | AAAAAGTCGACCCAGACCTAACCCACACACC |  |
| *mhpA*::NotI_cs_F1 | AAAAACTGCAGGTTTTCTCACCCCGAAAGG | Crossover PCR to make a fragment for constructing pDL6962 to insert NotI_cs_ between the palindrome and 3kb 3xChi array on the OD side, inside the *mhpA* gene |
| *mhpA*::NotI_cs_R1 | ACGTACCG**GCGGCCGC**AGC  GCGGCAGAAACATACGG |  |
| *mhpA*::NotI_cs_F2 | GCCGCGCT**GCGGCCGC**CGGTACGTCGCTTTGAATTT |  |
| *mhpA*::NotI_cs_R2 | AAAAAGTCGACAAGCGCATTTCGAGGAAGTA |  |
| *lacZY*:: NotI_cs_F1 | AAAAACTGCAGATTACTGCGACGGCTGACTT | Crossover PCR to make a fragment for constructing pDL7015 to insert NotI_cs_ between the 3kb 3xChi array on the OP side and the palindrome, in the *lacZY* region |
| *lacZY*:: NotI_cs_R1 | CGCTACCA**GCGGCCGC**TGGTCTGGTGTCAAAAATAA |  |
| *lacZY*:: NotI_cs_F2 | CCAGACCA**GCGGCCGC**TGGTAGCGACCGGCGCTC |  |
| *lacZY*:: NotI_cs_R2 | AAAAAGTCGACCCGGATTGATGGTAGTGGTC |  |
| *codA*.*cynR*:: NotI_cs_F1 | AAAAACTGCAGTTCATCTGCAAGGACGTTTC | Crossover PCR to make a fragment for constructing pDL6966 to insert NotI_cs_ between *codA* and *cynR* genes, 9kb away from the palindrome on the OP side |
| *codA.cynR*:: NotI_cs_R1 | CCCAGTCG**GCGGCCGC**TTCAACGTTTGTAATCGATG |  |
| *codA.cynR*:: NotI_cs_F2 | ACGTTGAA**GCGGCCGC**CGACTGGGTTACAGCGAGCT |  |
| *codA.cynR*:: NotI_cs_R2 | AAAAAGTCGACCCGCCACTACTGGAGAGAAC |  |
| *mhpE.mhpT*:: NotI_cs_F1  *mhpE.mhpT*:: NotI_cs_R1 | AAAAACTGCAGCCGAGAACGTGCATTTTGTA  CGGGCGAA**GCGGCCGC**ATGTAGGCCGGACAAGACGT | Crossover PCR to make a fragment for constructing pDL6964 to insert NotI_cs_ between *mhpE* and *mhpT* genes, 9kb away from the palindrome on the OD side |
| *mhpE.mhpT*:: NotI_cs_F2 | GCCTACAT**GCGGCCGC**TTCGCCCGTAGGCAGTCATT |  |
| *mhpE.mhpT*:: NotI_cs_R2 | AAAAAGGATCCCAAAGACCAGTGAGGGGAAA |  |
| *prpC*::NotI_cs_F1 | AAAAACTGCAGCATCCGACAACAATCTCGAC | Crossover PCR to make a fragment for constructing pDL7177 to insert NotI_cs_ within the *prpC* gene, 15kb away from the palindrome on the OP side |
| *prpC*::NotI_cs_R1 | GCCGGTAA**GCGGCCGC**GGCTTTCAGTTTCGTTTTGT |  |
| *prpC*::NotI_cs_F2 | TGAAAGCC**GCGGCCGC**TTACCGGCTAACGTGCGTAC |  |
| *prpC*::NotI_cs_R2 | AAAAAGTCGACCGTACAGCACCAGCGAGATA |  |
| *yaiX*::NotI_cs_F1 | AAAAACTGCAGGCCCCTGGGAAAGATTAAAA | Crossover PCR to make a fragment for constructing pDL7209 to insert NotI_cs_ within the *yaiX* gene, 15kb away from the palindrome on the OD side |
| *yaiX*::NotI_cs_R1 | TGGGCGAA**GCGGCCGC**CCGAACACGCAACTTGGCCC |  |
| *yaiX*::NotI_cs_F2 | GTGTTCGG**GCGGCCGC**TTCGCCCAGGCAAAATAATA |  |
| *yaiX*::NotI_cs_R2 | AAAAAGTCGACATTCAGGGGGCCAGTCTAGT |  |
| *prpR*::NotI_cs_F1 | AAAAACTGCAGAAAAAGGGGCTGATGTGTTG | Crossover PCR to make a fragment for constructing pDL7245 to insert NotI_cs_ within the *prpR* gene, 18kb away from the palindrome on the OP side |
| *prpR*::NotI_cs_R1 | CACTACGA**GCGGCCGC**GCAATATTCGTGAACTGCGC |  |
| *prpR*::NotI_cs_F2 | AATATTGC**GCGGCCGC**TCGTAGTGCAGCAGCACAGT |  |
| *prpR*::NotI_cs_R2 | AAAAAGTCGACCTATGAGGAAGGGGCGTTTA |  |
| *yaiS*::NotI_cs_F1 | AAAAACTGCAGTCATGCAATTTGCTCAAAGAA | Crossover PCR to make a fragment for constructing pDL7213 to insert NotI_cs_ within the *yaiS* gene, 18kb away from the palindrome on the OD side |
| *yaiS*::NotI_cs_R1 | TGCTGACA**GCGGCCGC**CATTTACAGCTCAATGATAT |  |
| *yaiS*::NotI_cs_F2 | TGTAAATG**GCGGCCGC**TGTCAGCAAAATTAAGATGA |  |
| *yaiS*::NotI_cs_R2 | AAAAAGTCGACGAACCCACGGGTCTGAAAG |  |
| *yahI.yahJ*::NotI_cs_F1 | AAAAACTGCAGCGCTGATCCAACAAGGTTTT | Crossover PCR to make a fragment for constructing pDL7246 to insert NotI_cs_ between *yahI* and *yahJ* genes, 24kb away from the palindrome on the OP side |
| *yahI.yahJ*::NotI_cs_R1 | CTTTCATC**GCGGCCGC**TTACGTTTTAATAATATGAG |  |
| *yahI.yahJ*::NotI_cs_F2 | AAACGTAA**GCGGCCGC**GATGAAAGAAAGCAATAGCC |  |
| *yahI.yahJ*::NotI_cs_R2 | AAAAAGTCGACATCATGTCCTGGATGGTGGT |  |
| *yaiT*::NotI_cs_F1 | AAAAACTGCAGGAGTTTGGTTGCGCTGCTAT | Crossover PCR to make a fragment for constructing pDL7214 to insert NotI_cs_ within the *yaiT* gene, 24kb away from the palindrome on the OD side |
| *yaiT*::NotI_cs_R1 | TATCGATA**GCGGCCGC**GGTATCATAGTTTGCTGCAT |  |
| *yaiT*::NotI_cs_F2 | ATGATACC**GCGGCCGC**TATCGATAATCCCGTTACAG |  |
| *yaiT*::NotI_cs_R2 | AAAAAGTCGACCAGCAACGCCTTCACTTGTA |  |
| *yahC.yahD*::NotI_cs_F1 | AAAAACTGCAGTAACGTCACCGTCCAGTTCA | Crossover PCR to make a fragment for constructing pDL7247 to insert NotI_cs_ between *yahC* and *yahD* genes, 30kb away from the palindrome on the OP side |
| *yahC.yahD*::NotI_cs_R1 | TGTTTCTT**GCGGCCGC**TACCCTTTCACTCAATCAGG |  |
| *yahC.yahD*::NotI_cs_F2 | AAAGGGTA**GCGGCCGC**AAGAAACAAATATTGCACTA |  |
| *yahC.yahD*::NotI_cs_R2 | AAAAAGTCGACCGGGTTTAGCCGGTAAAATA |  |
| *ampH.sbmA*::NotI_cs_F1 | AAAAACTGCAGCTGGTATGGGTTGCCAGATT | Crossover PCR to make a fragment for constructing pDL7215 to insert NotI_cs_ between *ampH* and *sbmA* genes, 30kb away from the palindrome on the OD side |
| *ampH.sbmA*::NotI_cs_R1 | TTGCAATC**GCGGCCGC**AGTAGTATAAATACGCTCAG |  |
| *ampH.sbmA*::NotI_cs_F2 | ATACTACT**GCGGCCGC**GATTGCAAAGGGCGAATTAG |  |
| *ampH.sbmA*::NotI_cs_R2 | AAAAAGTCGACAAATCCAACGACCAGAAACG |  |
| *ydeJ*::NotI_cs_F1 | AAAAACTGCAGCCGCGATAGCCATCTTCTAT | Crossover PCR to make a fragment for constructing pDL7565 to insert NotI_cs_ in *ydeJ* for the generation of a 11.6kb fragment to identify the 2n spot by 2-D gel |
| *ydeJ*::NotI_cs_R1 | TTTCAATA**GCGGCCGC**ATCTACAAGTTGTACAATTT |  |
| *ydeJ*::NotI_cs_F2 | TTGTAGAT**GCGGCCGC**TATTGAAAACCTGACATCCG |  |
| *ydeJ*::NotI_cs_R2 | AAAAAGTCGACGCCAGCCTGGTTATAGCAGT |  |
| *lacZ.*F | GCCCGGATAAACGGAACTGG | To generate a 958 bp fragment to be used as a template for making a Southern probe within *lacZ* between the two Chi arrays |
| *lacZ.*R | CTGCACACCGCCGACGGCAC |  |
| *cynR.cynS*.F | GCAATCCCAACGTCCAACTC | To generate a 1030bp fragment to be used as a template for making a Southern probe within the 3-9kb OP fragment from the palindrome |
| *cynR.cynS*.R | CCAGGCGCACCGATGGATGA |  |
| *mhpB*.F | CACTGTCTTTCCCACTCGCC | To generate a 929bp fragment to be used as a template for making a Southern probe within the 3-9kb OD fragment from the palindrome |
| *mhpB*.R | CAGTTCTCTGTTCTGGCGCT |  |
| *prpD*.F | TAACATCGGCGCGATGATCC | To generate a 1152bp fragment to be used as a template for making a Southern probe within the 9-15kb OP fragment from the palindrome |
| *prpD*.R | ACCGGCATCTGTTCCAGGCG |  |
| *adhC*.F | GCTGCCGTTGCATTTGCTCC | To generate a 969bp fragment to be used as a template for making a Southern probe within the 9-15kb OD fragment from the palindrome |
| *adhC*.R | AACCATGCCCGGTAACTGGG |  |
| *yahK*.F | GCTGTTGGTGCATATTCCGC | To generate a 1000bp fragment to be used as a template for making a Southern probe within the 18-24kb OP fragment from the palindrome |
| *yahK*.R | ATTTCACATCACCGCGCAGC |  |
| *tauB*.F | CGCCGATTATGGCGGCAAAC | To generate a 1051bp fragment to be used as a template for making a Southern probe within the 18-24kb OD fragment from the palindrome |
| *tauB*.R | CGATCCCGACCGGAATACCG |  |
| *yahF*.F | CTGATGTCTATCTCCACGCG | To generate a 1075bp fragment to be used as a template for making a Southern probe within the 24-30kb OP fragment from the palindrome |
| *yahF*.R | CCGGATCGGGCTGAATGTTG |  |
| *ampH*.F | TTTCTGCCGTGCTGTGTGCG | To generate a 1063bp fragment to be used as a template for making a Southern probe within the 24-30kb OD fragment from the palindrome |
| *ampH*.R | TTAAAGCGCGTCAACGGCGA |  |
| *ydeA*.F | CGCATGGGTAGTAGCGCTAA | To generate a 1016bp fragment to be used as a template for making a Southern probe to detect a 11.6kb fragment to identify the 2n spot by 2-D gel |
| *ydeA*.R | GCGTCTGTTCTTCGAGTGTC |  |
| pKO.F^a^ | AGGGCAGGGTCGTTAAATAGC | PCR to check the presence of a ligated fragment between the SalI and PstI sites into pTOF24 |
| pKO.R^a^ | AGGGAAGAAAGCGAAAGGAG |  |
| Δ*ruvAB*.F^c^ | GATCCCGACGTGATTACTCC | PCR to check *ruvAB* deletion by pDL2757 |
| Δ*ruvAB*.R^c^ | TGACGATTGGTGTAGCGATG |  |
| Δ*radA*.F^d^ | AAAAACTGCAGGATTGCCTACCATGCCAAG | PCR to check *radA* deletion by pDL4428 |
| Δ*radA*.R^d^ | AAAAAGTCGACTGCTTGATGGCAGTTTTCAG |  |
| Δ*recQ*.F^b^ | AAAAACTGCAGATGTGGTGGGTAATACTGACG | PCR to check *recQ* deletion by pDL2765 |
| Δ*recQ*.R^b^ | AAAAAGTCGACTTTTCAGTGCACCACGTAGC |  |
| *priA300*.F^a^ | AAAAACTGCAGTTTCCTGCTTCTTCGCTTTC | PCR to check *priA*300 base change by pDL4947 |
| *priA300*.R^a^ | AAAAAGTCGACATCTCTCGCTCATCGCAAAG |  |
| Δ*xonA.F*^b^  Δ*xonA.R*^b^ | AAAAACTGCAGAACCCGTCATCAGCTTTGTC  AAAAAGTCGACGCTGGATTGGCCTTGTATTT | PCR to check *xonA* deletion by pDL2745 |
| Δ*recJ.F*^b^  Δ*recJ.R*^b^ | AAAAACTGCAGTGGTTACTGCCACAAACTG  AAAAAGTCGACAAGGTTTCTTCGTCGTCAG | PCR to check *recJ* deletion by pDL2713 |
| *mhpA*::ChiF1 | AAAAACTGCAGAATTCAACAGCCAGGAAACG | To insert triple Chi array at the *mhpA* junction |
| *mhpA*::ChiR1 | CGCCATGTGACCACCAGCGAGTCTGCGCCCACCAGCGCGCAAGCTGTTAAGCAAAG |  |
| *mhpA*::ChiF2 | GCGCAGACTCGCTGGTGGTCACATGGCGGCTGGTGGAAATTCAAAGCGACGTACCG |  |
| *mhpA*::ChiR2 | AAAAAGTCGACGCCGTAACGCCTTTATTCAG |  |
| *lacZY*::ChiF1 | AAAAACTGCAGATTAGGGCCGCAAGAAAACT | To insert triple Chi array at the *lacZY* junction |
| *lacZY*::ChiR1 | CGATGCTGAGCTGGTGGACACGCGCTGGCTGGTGGTTATTTTTGACACCAGACCAACTG |  |
| *lacZY*::ChiF2 | CAGCGCGTGTCCACCAGCTCAGCATCGACCACCAGCATTTCGCGTAAGGAAATCCA |  |
| *lacZY*::ChiR2 | AAAAAGTCGACCACCGGCGTTAAAACAAAAG |  |

a – oligonucleotides designed by Dr Julia Mawer, Leach lab, b – oligonucleotides designed by Ewa Okely, Leach lab, c – oligonucleotides designed by Dr Martin White, Leach lab, d – oligonucleotides designed by Dr Charlie Cockram, Leach lab. OP means origin proximal and OD means origin distal. Cutting sites for PstI and SalI enzymes are underlined; Cutting sites for NotI enzyme are in bold.

**The sequence of triple Chi array at the MhpA junction**

**GCTGGTGG**GCGCAGACTC**GCTGGTGG**TCACATGGCG**GCTGGTGG**

**The sequence of triple Chi array at the LacZ/Y junction**

**CCACCAGC**CAGCGCGTGT**CCACCAGC**TCAGCATCGA**CCACCAGC**
